# A Validated Multiscale In-Silico Model for Mechano-sensitive Tumour Angiogenesis and Growth

**DOI:** 10.1371/journal.pcbi.1005259

**Published:** 2017-01-26

**Authors:** Vasileios Vavourakis, Peter A. Wijeratne, Rebecca Shipley, Marilena Loizidou, Triantafyllos Stylianopoulos, David J. Hawkes

**Affiliations:** 1 University College London, Centre for Medical Image Computing, Department of Medical Physics & Biomedical Engineering, London, United Kingdom; 2 University College London, Department of Mechanical Engineering, London, United Kingdom; 3 University College London, Department of Surgery, London, United Kingdom; 4 University of Cyprus, Department of Mechanical & Manufacturing Engineering, Nicosia, Cyprus; University of California San Diego, UNITED STATES

## Abstract

Vascularisation is a key feature of cancer growth, invasion and metastasis. To better understand the governing biophysical processes and their relative importance, it is instructive to develop physiologically representative mathematical models with which to compare to experimental data. Previous studies have successfully applied this approach to test the effect of various biochemical factors on tumour growth and angiogenesis. However, these models do not account for the experimentally observed dependency of angiogenic network evolution on growth-induced solid stresses. This work introduces two novel features: the effects of hapto- and mechanotaxis on vessel sprouting, and mechano-sensitive dynamic vascular remodelling. The proposed three-dimensional, multiscale, in-silico model of dynamically coupled angiogenic tumour growth is specified to in-vivo and in-vitro data, chosen, where possible, to provide a physiologically consistent description. The model is then validated against in-vivo data from murine mammary carcinomas, with particular focus placed on identifying the influence of mechanical factors. Crucially, we find that it is necessary to include hapto- and mechanotaxis to recapitulate observed time-varying spatial distributions of angiogenic vasculature.

## Author Summary

Angiogenesis is a hallmark of tumour growth and a key feature for invasion and metastasis. Thus, to elucidate the governing biological processes of cancer development, it is instructive to develop physiologically representative mathematical and computational (in-silico) models with which to compare to experimental data. We present a validated, three-dimensional, in-silico model of tumour-induced angiogenesis and growth—unique in the field of mathematical cancer modelling in its biophysical prescription and predictive accuracy. Our novel modelling approach encompasses the effects of both biochemical and biomechanical processes on the development of angiogenic vasculature and solid tumour growth. Unlike other works—which typically focus on biochemical processes—our model depicts a dynamic and coupled biophysical system of the tumour-host microenvironment. In concert with in-vivo experimental data, we have a physiologically-representative in-silico model that enables us to classify and rank the governing biological processes. This yielded the remarkable discovery that it is necessary to include biomechanical factors to accurately describe angiogenic topology. Our model not only provides insight into fundamental cancer biophysics, but is also a powerful tool to aid in our overall objective of re-engineering the tumour microenvironment to optimise the delivery of therapeutic drugs.

## Introduction

The role of angiogenesis—the process whereby existing blood vessels produce new vasculature—in cancerous growth, invasion and metastasis has been extensively studied over the past five decades. Starting with the assertion of Folkman [1] that angiogenesis is a necessary component for neoplasmic growth, the current paradigm is that tumours induce neo-vascularisation upon reaching an avascular limit [2]. This limit represents a critical tumour size that can be supported by oxygen diffusion from the existing vasculature alone, beyond which substrate gradients produce internal regions of oxygen deprivation, i.e. hypoxia.

To avoid necrosis, cells in the hypoxic regions secrete diffusible chemical signals, termed tumour angiogenic growth factors (TAFs). Upon reaching the existing vasculature, the TAFs stimulate endothelial cells (ECs) to degrade their basement membrane and extracellular matrix (ECM) via the secretion of matrix metalloproteases (MMPs) [1]. Motile ECs then migrate from the vessel lining up the TAF gradient field towards the TAF source, forming tubes with sprout-tips at the leading edge of a new vascular lumen. These tubes can form networks in a process termed anastomosis and penetrate into the tumour, depending on how the ECs respond to mechano-chemical factors. As blood flows through the vessels and remodels their structure through its response to fluid shear stresses and vascular pressure, the tumour is provided with a direct supply of nutrients and oxygen, enabling further expansion into the surrounding tissue. The neo-vasculature, however, is pathological and during tumour growth its structural integrity can be compromised. Indeed solid stresses in the tumour are elevated as a consequence of rapid growth into the confined space of the host tissue, which can compress and ultimately collapse intra-tumoural blood vessels, rendering tumours hypo-vascular and hypo-perfused [3, 4]. Hypo-perfusion, in turn, has been shown to inhibit the delivery of chemotherapy, reducing drastically treatment efficacy [5, 6].

Furthermore, a key factor in angiogenic tumour growth is cell response to mechano-chemical cues. Of particular interest is their directed motion along chemical gradients, termed chemotaxis and haptotaxis for soluble (e.g. oxygen) and insoluble (e.g. proteoglycan) substrates, respectively, and mechanical gradients (e.g. solid stresses), termed mechanotaxis. This produces a biophysical system with multiple components interacting at multiple scales. In order to study the effect of a given component and characterise its physical origins, it is instructive to construct physiologically-representative mathematical models. Here we focus on continuum models of tumour growth coupled with angiogenesis; for more detail on angiogenesis modelling alone, see the recent review by Scianna et al. [7]. Whereas previous studies have explored the chemical, i.e. solute-driven, underpinning of both tumour growth and angiogenesis, here we focus on the interplay between angiogenic network evolution and growth-induced solid stress generation.

Broadly speaking, the tumour and vasculature can each be described by continuum, discrete or hybrid models, with their coupling either static or dynamic. One approach is to characterise the angiogenic response in terms of the blood vessel density, with the dynamics and chemical factors obeying continuum conservation laws. Prominent early examples are [8, 9], which extended the seminal work of Balding and McElwain [10] to define a set of coupled integro-differential equations that characterise tumour-induced neo-vascularisation and network formation. More recently in [11], the authors utilised a continuum approach to investigate the role of feedback regulation processes on sprout inception. However, the blood vessel density paradigm is unable to account for vascular morphology and its explicit impact on blood flow heterogeneity.

In order to characterise network morphology and blood flow, it is necessary to model the vasculature discretely in terms of line segments, curves or lattices. This approach was adopted by Zheng et al. [12], who employed a hybrid model to describe the vasculature [13] coupled with a nonlinear continuum description of the tumour mass [14]. The model simulated physiologically-realistic tumour morphologies as a result of a static coupling with angiogenic growth, but was limited in its description of tumour-environment interactions and mechanical factors. This model was later extended by Macklin et al. [15] to include dynamic angiogenesis, allowing for an explicit description of vascular remodelling and blood flow, and further extended by Wu et al. [16] to model the effects of interstitial fluid pressure. A contrasting approach was proposed by [17], who presented a solid mechanics description of the tumour and its environment. Their model featured a dynamic coupling of angiogenesis and vascular remodelling with a growing domain, and accounted for deformation due to growth, response to hypoxia and blood flow. However, there was no account of the effect of solid stress on vascular development and integrity inside the tumour.

To account for the effect of haptotactic (i.e. insoluble) vascular endothelial growth factors (VEGF), Milde et al. [18] presented a deterministic, hybrid model of sprouting angiogenesis. The matrix-bound VEGF was cleaved by MMPs produced at the endothelial tip cells, and the response characterised in terms of the resulting vessel geometries. Furthermore, they included the effect of ECM fibre density and structure on the sprout tip migration velocity. They found that high density matrices produced shorter capillary networks, and observed an increase in the number of branches with the density of matrix-bound VEGF. Similar results were obtained by Bauer et al. [19], who developed a cell-based model of tumour-induced angiogenesis. In both models, however, there was no account of mechanotaxis or the effects of vessel wall collapse due to applied solid stresses. Previous studies by Breward et al. [20] and Bartha et al. [21] have presented models describing the effect of pressure on vessel integrity, but no attempt is made to explicitly model vessel compression owing to intratumoural forces.

To our knowledge there is no existing model that dynamically couples capillary growth, morphology and structure with mechanochemically-regulated blood flow and growth-induced solid stresses. Numerical simulations of angiogenic tumour growth are performed for spheroidal geometries, which we use to gain insight into the relationship between mechano-chemical factors and tumour development. Key measures of vascular development, defined in the following section, are validated using data from in-vivo murine mammary carcinomas [22]. The hypotheses we test are:

1. The model can quantitatively reproduce observations of vascular density and distribution in pathological tissues.
2. The model can qualitatively reproduce observations that growth-associated stress influences vessel morphology and structural integrity.

One part of the novelty of the mathematical formulation lies in the introduction of a mechanotactic term to the function defining the orientation of capillary tip elongation. The second part is the use of a phenomenological description of the tip extension velocity. The final part is a set of constitutive terms defining the capillary wall remodelling as a function of mechanical factors. These features furnish the model with a complete, if simplified, description of the biophysical factors influencing tumour-induced angiogenesis.

Specifically, a discrete three-dimensional model of vascular sprouting is employed to describe the angiogenic response to TAF secretion, where the sprout-tips are represented as point masses in a continuum substratum [23]. The secretion of TAFs and MMPs by the tumour and vasculature, respectively, are described by coupled reaction-diffusion equations [24]. Tumour growth is modelled according to a Gompertz-type relation derived in previous work [25], and the quasi-static linear momentum equation is solved at the macroscopic scale assuming hyperelastic material properties. Capillary elongation, branching and remodelling are made dependent upon mechanical factors, such as traction and magnitude of wall shear stress. Finally, similar to Stylianopoulos and Jain [26], intra-, trans- and extravascular fluid flow is described by Poiseuille’s, Starling’s and Darcy’s laws respectively. To our knowledge, we present here for the first time a validated, three-dimensional, tumour-induced angiogenesis model using published in-vivo data. These data include the vascular density and structure obtained from image analysis of MCaIV carcinomas. To constrain the model, ex-vivo measurements of MCaIV carcinoma material properties were used as input parameters. Where possible all other input parameters were specified according to either in-vivo or in-vitro data from the literature.

## Materials and Methods

**Fig 1:**
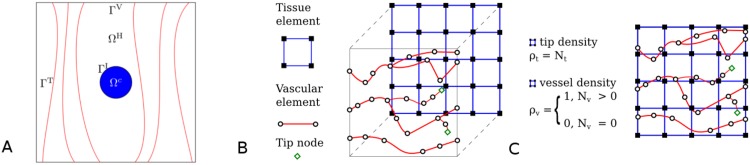
Schematics of the domain of analysis: the discretised tissue domain and the vascular network. Schematics of the domain of analysis: the discretised tissue domain and the vascular network. A: Schematic representation of the tumour–host domain, from inside out: solid-tumour (Ω^T^) and healthy tissue (Ω^H^) containing a network of capillaries. B: Graphical illustration of a representative grid of tissue elements (shown here using two-dimensional finite elements) with the one-dimensional grid of the vascular tree projected on top. Black squares represent the tissue element nodes, and hollow black circles the vascular element nodes. The hollow green diamonds represent vascular network end-points (i.e. tip ECs). C: An overlay of the two meshes, showing the tip and functional vascular-node density, *ρ*_t_ and *ρ*_v_, respectively. Note that the finite element mesh of the tissue domain is non-conforming to the corresponding finite element mesh of the vascular network (see also S1 Fig).

Let us denote the volume of the tumour by Ω^T^ and that of the host (healthy) tissue by Ω^H^, such that Ω = Ω^T^ ∪ Ω^H^ is the total volume of the biological tissues involved (macroscopic level). The analysis domain Ω is bounded by Γ = Γ^V^ + Γ^T^, where Γ^V^ are surfaces with vessel inlets or outlets, Γ^T^ are surfaces with no vessel inlets or outlets, and the tumour–host interface boundary is denoted by Γ^I^. The boundary Γ is set sufficiently distant from the tumour region to avoid the imposed boundary conditions impacting the solution in the tissue volume of interest. Fig 1A shows a schematic representation of the analysis domain.

The equations that describe the balance of the various biophysical species are expressed in a Lagrangian frame of reference. Hence the Laplacian operators used to express Fickian diffusion are expressed with respect to the initial (reference) setting: $\left\{X\in \Omega \;|\;\Omega \subset R^{3}\right\}$. Whilst the vasculature is treated separately, the surrounding interstitium, comprised of cells and matrix, is treated as one phase with uniform properties; inertial terms corresponding to interstitial fluid flow are neglected for simplicity, given the small Reynolds number for these flows compared to vascular transport (for example). The strong-form equations are then transformed into the corresponding weak-form, and subsequently discretised using the conventional finite element (FE) method. Time-integration of the biochemical solver module is achieved using an explicit numerical scheme (forward Euler method), whereas the solid solver module, which incorporates the growth model, utilises a full-implicit iterative (Newton-Raphson) scheme. More details are provided in the Solution strategy: Coupled multiscale solver subsection.

A schematic illustration of the discretisation method of the tissue and the vascular-network domain is shown in Fig 1B. It demonstrates that the two finite element meshes are non-conforming, where the 1D vascular network is contained in the 3D grid of the tissues (either healthy or pathological) without sharing any nodes. The two meshes are superimposed in Fig 1C. Here, two vascular network properties with respect to the tissue grid are defined: the tip density, *ρ*_t_, which is equal to the number of tip vascular nodes, N_t_, contained in a tissue element; and the vascular density *ρ*_v_. N_v_ is the number of well-perfused vascular nodes contained in a tissue element. Definition of a well-perfused vascular node is explicitly provided in the Capillary wall remodelling subsection. Thus, in the initial state (*t* = 0), the vasculature is uniformly distributed in the healthy tissue with all inlets and outlets confined to two opposite planes.

The following section details the construction of the solid, biochemical, vascular and fluid models. All variables (i.e. oxygen concentration, metalloproteinases concentration, etc.) are normalised relative to typical values for ease of presentation; these normalisation scales, and all parameter values within the models are given in the Supporting Information. We present the model equations for each (solid, biochemical, vascular, fluid) model first, and suspend assigning initial and boundary conditions for the model until the Initial and boundary conditions subsection.

### Tissue biomechanics

#### Extracellular matrix structural model

The structural integrity and composition of the extracellular matrix (ECM) of the host tissue is assumed to change in time. It is well known that the cleaving of the ECM is a crucial step in EC migration—and hence angiogenesis—which is mediated by a group of proteins known as matrix metalloproteinases (MMPs) [27], also referred to as matrix-degrading enzymes. We describe structural changes at the stroma of the host tissue using a first-order ordinary differential equation for the ECM density, *ϵ*, that accounts for degradation of the matrix due to the presence of matrix-degrading enzymes, *μ*, in the interstitium [28].

$$\frac{d\epsilon }{dt}=-\;\delta_{\epsilon }\;\mu \;\epsilon \;,\;\forall \;X\in \Omega^{\text{H}}\;,$$ (1)

where *δ*_*ϵ*_ is the ECM degradation rate (given in days^-1^), while “*d*./*dt*” denotes the material derivative (= ∂./∂*t* + **v** ⋅ ∂./∂**x**; **v** the velocity in spatial coordinates of a material point in the ECM). A detailed description of the mathematical model for the *μ* state variable is given in the following subsection.

#### Tissue solid biomechanics model

Using quantities related to a reference configuration of the analysed domain—defined here as an avascular tumour embedded in a vascularised extracellular matrix (which is the setup at *t* = 0), as in Fig 1A—equilibrium of the biological tissues (tumour and host) can be described by the Navier-Cauchy equation. In this reference state, initial pre-stresses are assumed negligible compared to the subsequent increase of solid stresses and fluid pressure in the interstitium during vascular cancer growth. Thus, the linear momentum equation in a Lagrangian framework is given by

$$\frac{\partial }{\partial X}\cdot \left[F\cdot S\right]+\rho \;b=0\;,\;\forall \;X\in \Omega \;,$$ (2)

where **S** is the second Piola-Kirchhoff stress tensor, *ρ* is the mass density of the tissues involved in the reference setting, and **b** a body force vector per unit of mass. Here both inertial and body forces are considered negligibly small compared to the internal stresses produced by large strains, and are hence eliminated. The former is justified by considering that, assuming an approximately constant cell velocity, the tissue acceleration is approximately zero. Body forces are neglected by assuming no external excitation, hence **b** = 0. In addition, viscous forces are also considered negligible due to the low tissue velocities modelled in this analysis: an approximate increase in diameter of 160 *μ*m day^-1^.

Following the continuum mechanics theory of soft tissue growth in biomechanics, originally proposed by Fung [29], we assume a multiplicative decomposition of the deformation gradient tensor into an inelastic (growth/irreversible) part, **F**_g_, and an elastic (reversible) part, **F**_e_. Thus, the deformation gradient tensor, **F**, can be expressed as [25]: **F** = **F**_e_ ⋅ **F**_g_, where **F**_g_ = λ_g_ **I**, with **I** the identity tensor and λ_g_ the volumetric stretch ratio, related to the Green-Lagrange volume strain through $\vartheta_{\text{g}}=\left(\lambda_{\text{g}}^{2}-1\right)/2$; note that this assumes isotropy of tissue remodelling. In the present study, permanent volumetric deformation is expressed phenomenologically with respect to the concentration of oxygen, *ξ* ∈ [0, 1], in the cancer mass via an exponential growth function:

$$\vartheta_{\text{g}}=\alpha_{\text{g}}\;\text{exp}\left[-\beta_{\text{g}}\;\text{exp}\left[-\gamma_{\text{g}}\;\xi \right]\right]-\alpha_{\text{g}}\;\text{exp}\left[-\beta_{\text{g}}\right]\;,$$ (3)

where *α*_g_, *β*_g_ and *γ*_g_ are dimensionless growth parameters, while oxygen concentration is a state variable numerically evaluated from the system (see Eq (13)). Note that the volumetric strain is thus implicitly dependent on time via the concentration of oxygen.

This work extends the definition of **F**_g_ to account for the effect of intra-tumoural local gradients of oxygen concentration, **∇***ξ*, and ECM density, **∇***ϵ*, on growth. It is assumed that the nominal strains in the direction of the respective gradients, *ε*_g-*ξ*_ and *ε*_g-*ϵ*_, obey a similar growth function as Eq (3) with respect to *ξ* but with different parameter values. However, following Lubarda and Hoger [30], for the general case of non-isotropic growth the deformation gradient tensor can be expressed as: **F**_g_ = λ_g_ **I** + (λ_g-*ξ*_ − λ_g_)**∇***ξ* ⊗ **∇***ξ* + (λ_g-*ϵ*_ − λ_g_)**∇***ϵ* ⊗ **∇***ϵ*, where λ_g-*ξ*_ and λ_g-*ϵ*_ are the corresponding stretch ratios, and the dyadic operator “⊗” denotes a tensor product. Having computed the inelastic deformation gradient, the elastic deformation gradient tensor is returned via: $F_{\text{e}}=F\cdot F_{\text{g}}^{-1}$, with the Green-Lagrange elastic strain given by $E_{\text{e}}=\left(F_{\text{e}}^{T}\cdot F_{\text{e}}-I\right)/2$. Finally, making use of the above constitutive equation, the mechanical stress tensor can be evaluated at any point of the tumour–host domain of analysis.

Both the tumour and the host tissue are modelled as a non-viscous, non-linear, hyperelastic continuous medium that can undergo large deformations and rotations [25, 31]. The constitutive description of the soft tissue biomechanics is given by the general form constitutive equation [32]: $S=\partial \bar{W}/\partial E_{\text{e}}$, where **E**_e_ the Green-Lagrange elastic strain, as explained below, and $\bar{W}$ is a potential function—also referred in the literature as stored-energy function—which $\bar{W}>0$ and is typically expressed with respect to the invariants of tensor **E**_e_ [32]. In this work, we describe both the tumour and the host tissue using a generalised polynomial expression of the stored-energy function: $\bar{W}=c_{10}\;\left({\bar{I}}_{1}-3\right)+c_{20}\;{\left({\bar{I}}_{1}-3\right)}^{2}+c_{01}\;\left({\bar{I}}_{2}-3\right)+c_{02}\;{\left({\bar{I}}_{2}-3\right)}^{2}+c_{11}\;\left({\bar{I}}_{1}-3\right)\;\left({\bar{I}}_{2}-3\right)+\kappa \;{\left(J-1\right)}^{2}/2$, where *J* is the determinant of the elastic deformation gradient tensor, while ${\bar{I}}_{1}$ and ${\bar{I}}_{2}$ is the first and second invariant of the deviatoric part of the recoverable right Green-Cauchy tensor respectively [32]. The parameters *c*_*ij*_, *κ* are material constants, where the latter is approximately equal to the bulk modulus in the small deformation regime.

In order to effectively describe the coupling between the time-varying ECM density, due to the interaction of MMPs with insoluble species (e.g. collagen fibres), with the solid macro-mechanics of the host-tissue–we introduce a single factor describing the structural integrity of the ECM through isotropic damage to the matrix (c.f. the reduction factor defined in Holzapfel’s book [32], Chapter 6). An internal scalar variable, termed here integrity factor, takes values within the range *ζ* ∈ (0, 1] while it quantifies the magnitude of isotropic damage in the continuous hyperelastic solid (i.e. the degradation of the ECM). Let *ζ* describe the structural health condition of the matrix, therefore *ζ* is assumed to be equivalent to the extracellular space density, *ϵ*, in the host-tissue domain, Ω^H^. Thus, at the beginning of the cancer development simulation *ζ* = 1, ∀**X** ∈ Ω^H^ since *ϵ* = 1 is taken as an initial condition. However, during the course of the analysis the matrix-degrading enzymes concentration, *μ*, increases—owing to the secretion of MMPs by the tumour and the tip endothelial cells—thus leading to a local decrease of the ECM density, *ϵ*. The matrix integrity factor, *ζ*, is introduced into the constitutive equation through the stored-energy function; hence, isochoric mechanical stresses in the ECM are scaled by *ζ* albeit the volumetric part is left intact, i.e. $\bar{W}=\zeta c_{10}\;\left({\bar{I}}_{1}-3\right)+\zeta c_{20}\;{\left({\bar{I}}_{1}-3\right)}^{2}+\zeta c_{01}\;\left({\bar{I}}_{2}-3\right)+\zeta c_{02}\;{\left({\bar{I}}_{2}-3\right)}^{2}+\zeta c_{11}\;\left({\bar{I}}_{1}-3\right)\;\left({\bar{I}}_{2}-3\right)+\kappa \;{\left(J-1\right)}^{2}/2$. The material parameter values for the above constitutive equations are provided in S1 Table, including references to the relevant literature.

#### Blood and interstitial fluid flow model

We describe the haemodynamics in the individual capillaries using Poiseuille’s equation [29], where the volumetric flow rate in a vessel is related to the pressure drop across it via

$${\dot{Q}}_{\text{vsc}}=-\;\frac{\pi R^{4}\left(t\right)}{8\;\mu_{\text{B}}\;L_{\text{vsc}}\left(t\right)}\;\Delta p_{\text{vsc}}\;,$$ (4)

where *μ*_B_ the blood viscosity, *R* is the capillary lumen radius with cross-sectional area *πR*^2^, and *L*_vsc_ is the length of a capillary segment. Note that R and *L*_vsc_ change in time since vessels are deformed under solid stresses. The blood viscosity is assumed homogeneous and constant in time; this is in order to remove an additional model parameter in lieu of suitable data with which to inform it. Interstitial fluid flow is described using Darcy’s law [33, 34] so that the volumetric fluid flow rate in the extracellular space is given by

$${\dot{Q}}_{\text{int}}=-\;\frac{K_{\text{int}}\;A_{\text{int}}}{L_{\text{int}}\left(t\right)}\;\Delta p_{\text{int}}\;,$$ (5)

where *K*_int_ is the hydraulic conductivity of the interstitium, *A*_int_ is the interstitium cross-sectional area, and *L*_int_ is the length of a tissue segment whose interstitial fluid pressure difference is denoted by Δ*p*_int_. The cross-sectional area can be expressed with respect to the mean capillary radius and the vascular density, *S*_vsc_, as $A_{\text{int}}=2\pi \bar{R}/S_{\text{vsc}}$ [35]. Here $\bar{R}$ is the average capillary radius in the local neighbourhood of the connective tissues under consideration.

To model the fluid movement across the capillary barrier that occurs as a result of filtration, we use Starling’s equation. Similarly to Baish at al. [35], the volumetric transvascular flow rate across the permeable endothelium is expressed through Starling’s law

$${\dot{Q}}_{\text{trv}}=K_{\text{vsc}}\left(t\right)\;A_{\text{vsc}}\left(t\right)\;\left(p_{\text{eff}}-p_{\text{int}}\right)\;.$$ (6)

Here *K*_vsc_ is the hydraulic conductivity of the endothelial barrier, which can be expressed as a function of the size of the fenestrations on the vessel (pores’ average radius), *r*_p_, the fraction of vessel-wall surface occupied by pores, *γ*_p_, and the blood viscosity, *μ*_B_, via [36]:

$$K_{\text{vsc}}=\frac{\gamma_{\text{p}}\;r_{\text{p}}^{2}\left(t\right)}{8\;\mu_{\text{B}}\;h\left(t\right)}\;.$$ (7)

Finally, *A*_vsc_ is the surface area of the blood vessel wall and the “effective” pressure is given by

$$p_{\text{eff}}=p_{\text{vsc}}-\left(\pi_{\text{vsc}}-\pi_{\text{int}}\right)\;\sigma_{\text{o}}\;,$$ (8)

where *σ*_o_ is the average osmotic reflection coefficient of the plasma proteins, *π*_vsc_ is the osmotic pressure of the plasma at the permeable vascular wall, while *π*_int_ is the corresponding osmotic pressure of the interstitial fluid. This modelling approach accounts for the contribution of the colloid osmotic pressure of plasma and interstitial fluid. Including those features are important for a complete modelling description of the micro-circulation system. Nonetheless, numerical experiments have revealed that the omission of the rightmost term in Eq (8) in the vascular–interstitium interaction model has only marginally affected the qualitative predictions of the proposed tumour-growth angiogenesis model. This is also supported by the experimental findings of Tong and colleagues [37], who showed that the osmotic pressure difference across the wall of tumour vessels is negligible.

It is important to note here that we do not include the lymphatic system in the current model, given that it is generally assumed to be compromised in tumour tissues. However, this would be a straightforward extension to the current framework.

The flow rate Eqs (4)–(6) are coupled to a model for the vascular and interstitial pressures. In the vascular network, conservation of fluid flux at vessel junctions provides a linear system of equations to solve for the nodal pressures, subject to pressure/flow boundary conditions on the terminal nodal points of the network. The interstitial pressure, *p*_*int*_, satisfies the Poisson equation, where the source term captures both vascular and osmotic contributions, following the approach of Stylianopoulos and Jain [26]. Quasi-steady state fluid flow is solved numerically for the vascular and interstitial pressures (defined on nodal points in the vascular network, and extravascular-space points, respectively). An interconnected grid of tissue and vascular nodes is considered. Tissue nodes are connected to each other via the 3D FE mesh, where each tissue element corresponds to a two node edge element of the FE grid. Vascular nodes are connected to each other according to the network structure generated by the vascular network module, defined in the Angiogenesis model subsection. Also, as shown in the 2D illustration of Fig 1C, each vascular node is contained in a FE of the discrete three-dimensional domain of analysis. Thus, in order to describe transvascular flow through Eq (6), each vascular node is associated with the corresponding vertices (i.e. tissue nodes) of the FE.

After every simulation of the flow model, the magnitude of the average wall shear stress (WSS) distribution, *τ*_f_, the axial blood-flow mean velocity in a vascular segment, *v*_vsc_, and the fluid velocity at the interstitium, *v*_int_, can be evaluated using the following relationships:

$$\tau_{\text{f}}=R\;|\Delta p_{\text{vsc}}|\;/L_{\text{vsc}}\;,$$ (9)

$$v_{\text{vsc}}={\dot{Q}}_{\text{vsc}}\;/\pi R^{2}\;,$$ (10)

$$v_{\text{int}}=-K_{\text{int}}\;\Delta p_{\text{int}}\;/L_{\text{int}}\;,$$ (11)

where Δ*p*_vsc_ and Δ*p*_int_ is the pressure difference between two vascular and two interstitial nodes respectively of the corresponding discretised domains of analysis (i.e. the vascular network and the ECM) respectively. The value set for the material parameters of the above equations are provided separately in S2 Table.

### Tissue biochemical model

One of the first steps in angiogenesis is the production of diffusible angiogenic growth factors (such as VEGF, PDGF, etc.) by tumour cells, and their subsequent binding to corresponding receptors of nearby blood vessels [38]. For model simplicity, we focus on a single growth factor—referred here as the tumour-angiogenic factor (TAF)—as a homogenised chemical modulator of capillary sprouting and elongation. It would be straightforward to extend this model to account for multiple interacting growth factors (e.g. VEGF binding receptors and inhibitors) to represent the complex patho-physiology of tumour-induced angiogenesis. However, given that it is well-documented that interstitial fluid velocities are weak compared to vascular flow and diffusion [39], we have not included advection in the tissue biochemical transport model.

Transport of TAF (with normalised concentration denoted *τ*) is described by a reaction-diffusion equation [15] that accounts for random spatial diffusion, TAF production as a function of the normalised local oxygen concentration, *ξ*, in the tumour (defined below), and its natural decay and finally loss due to cellular consumption,

$$\frac{d\tau }{dt}=\frac{\partial }{\partial X}\cdot \left[D_{\tau }\;\frac{\partial \tau }{\partial X}\right]+Q-\delta_{\tau }\;\tau \;,\;\forall \;X\in \Omega \;,$$ (12)

where *D*_*τ*_ is the isotropic diffusion coefficient for TAF (given in m^2^ day^-1^), *δ*_*τ*_ represents the aggregate loss due both decay and cellular consumption, the production rate *Q* (given in day^-1^) is defined by

$$Q\left(\xi \right)=\left\{\begin{array}{cc} \;\lambda_{\tau }\;\text{exp}\left[-2\;\xi /\bar{\xi }\right]; & \text{if}\;X\in \Omega^{\text{T}}\\ \;0; & \text{elsewhere} \end{array}\right.\;,$$

where $\bar{\xi }$ is a scaling parameter that modulates the oxygen level at which cancer cells release TAF, and λ_*τ*_ is a TAF-production rate parameter. Parameter values are provided in S3 Table, including references to the relevant literature.

The microvascular network provides a uniform source of oxygen, which diffuses into the interstitial space and is consumed by the cells [28], such that

$$\frac{d\xi }{dt}=\frac{\partial }{\partial X}\cdot \left[D_{\xi }\;\frac{\partial \xi }{\partial X}\right]+\lambda_{\xi }\;\rho_{\text{v}}-\delta_{\xi }\;\xi \;,\;\forall \;X\in \Omega \;,$$ (13)

where *D*_*ξ*_ is the isotropic diffusion coefficient for oxygen (given in m^2^ day^-1^), λ_*ξ*_ is the oxygen production rate due to supply from the microvascular network and *δ*_*ξ*_ is the consumption rate of the species by the cancerous cells (both expressed in day^-1^). Here, the dimensionless parameter *ρ*_v_ represents the average vascular density in a tissue FE; if fully-functional, well-perfused blood vessels are present in a particular element then *ρ*_v_ = 1, whereas if the vessels are hypo-perfused or non-functional (i.e. collapsed) or absent then *ρ*_v_ = 0; see Fig 1B and 1C for a visual guide. Definition and differentiation of a well-perfused from a hypo-perfused node of the vascular network is explicitly provided in the Capillary wall remodelling subsection, while definition of a collapsed vascular node is given further in the Interaction between the tumour–host biomechanics and the capillaries subsection.

Finally, the concentration of the matrix-degrading enzymes: MMPs, *μ*, in the extracellular space of the host tissue is modelled through [15, 40]

$$\frac{d\mu }{dt}=\frac{\partial }{\partial X}\cdot \left[D_{\mu }\;\frac{\partial \mu }{\partial X}\right]+F-\delta_{\mu }\;\mu \;,\;\forall \;X\in \Omega^{\text{H}}\;.$$ (14)

Here *D*_*μ*_ (m^2^ day^-1^) is the isotropic diffusion coefficient for MMPs, while *δ*_*μ*_ (day^-1^) represents the natural decay of *μ*. The production rate function *F* of MMPs (day^-1^) is taken as the superposition of contributions of production from the proliferating cancer cells and the tip-endothelial cells, defined as:

$$F=\left\{\begin{array}{cc} \;\lambda_{\mu \text{-c}}+\lambda_{\mu \text{-v}}\;\rho_{\text{t}}; & \text{if}\;X\in \Omega^{\text{T}}\\ \;\lambda_{\mu \text{-v}}\;\rho_{\text{t}}; & \text{elsewhere} \end{array}\right.\;.$$

In a similar fashion to *ρ*_v_, the dimensionless variable *ρ*_t_ represents the density of tip vascular nodes in a finite element of the tissue domain, which is zero when no newly-formed sprouts are present and increases proportionally with the number of branches present (see Fig 1). Evidently, both *ρ*_v_ and *ρ*_t_ vary with respect to space since not all finite elements contain vascular segments or tip vessels, and also with respect to time due to the temporal evolution of the network structure as it undergoes angiogenesis.

As is evidenced by Eqs (12), (13) and (14), the diffusion coefficients are assumed to be constant and homogeneous everywhere (see S3 Table for the parameter values). The mathematical model of tumour-induced angiogenesis hence assumes linear, Fickian diffusion of the chemical species.

### Angiogenesis model

A description of the dynamic angiogenesis model is presented here. The model is decomposed into to two primary components: a model of capillary sprouting, and a model that couples capillary wall mechanics with growth-induced solid and fluid mechanical loads.

#### Capillary sprouting

The orientation vector of capillary sprout elongation—denoted by the unit vector $\hat{e}$—is defined by the linear superposition of the vectors of: (i) the chemotactic contribution due to TAF gradients, **∇***τ*; (ii) the haptotactic contribution due to insoluble ECM gradients, **∇***ϵ*; and (iii) the mechanotactic contribution due to mechanical stresses, ***t***.

$$\hat{e}=\ell \;/\;∥\ell ∥\;\text{;}\;\text{where}\;\;\ell =k_{\tau }\;\nabla \tau +k_{\epsilon }\;\nabla \epsilon -k_{\text{m}}\;t\;.$$ (15)

Here the scalar parameters *k*_*τ*_, *k*_*ϵ*_ and *k*_m_ represent the relevant importance of each taxis component in determining the capillary sprout elongation (see S4 Table for parameter values). The traction vector *t* is equal to the eigenvector related to the minimum eigenvalue of the 3 × 3 symmetric mechanical stress tensor, **S**. The stresses and the gradients of *τ* and *ϵ* at a point of the vascular network (vessel tip or branch) are computed by interpolating the computed gradient fields at the quadrature points of the FE that contains this vascular node. Note that linear additivity of each component is implicitly assumed to simplify the expression.

This description offers an extension to previous angiogenesis models in the literature. For example, the authors [23] describe vessel elongation using a discrete modelling approach similar to Eq (15) above, but account for chemotaxis only. However, Milde et al. [18] described the vascular elongation direction using a combination of chemo- and haptotactic cues in the extracellular space. Furthermore, McDougall et al. [40] modelled vessel sprouting using a continuum-based approach by considering both hapto- and chemotaxis for the endothelial cells migration in the interstitium. However, they do not incorporate mechanotaxis, which has been identified as playing an important role in endothelial cell kinematics: Li et al. [41] observed that cells migrate preferentially in the direction of fluid shear stress. These observations were also made by Lin et al. [42] using micropatterned substrates. More recently, and with particular relevance to our work, Edgar et al. [43] demonstrated the importance of mechanics in in-vitro angiogenesis via a coupled FE model of neo-vessel growth and ECM biomechanics.

**Fig 2:**
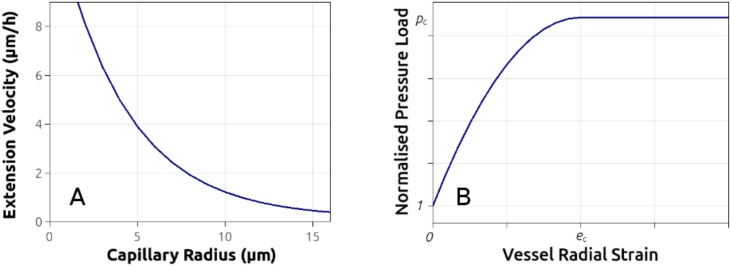
Capillary-tip extension velocity and vessel wall mechanical model. A: Extension rate of vascular-tip endothelial cells versus the capillary radius expressed by an exponential decay function, fitted to reported in-vitro angiogenesis experiments [44]. B: Stress–strain plot of the constitutive equation used to describe the biomechanics of the blood vessels, including the pressure which induces vessel collapse, *p*_c_.

Experimental observations made by Wood and his colleagues [44], obtained from an in-vitro angiogenesis microfluidics platform, indicate that tip elongation speed is inversely correlated with the radius of the vessel lumen. Following this description, the tip extension velocity, *v*_v_, is related to the capillary lumen radius, *R*, via the relationship

$$v_{\text{v}}=v_{\text{v-0}}+v_{\text{v-1}}\;\text{exp}\;\left[-R/\tilde{R}\right]\;,$$ (16)

where *v*_v-0_, *v*_v-1_ and $\tilde{R}$ are constants determined by fitting the above expression to measured data [44] (plotted in Fig 2A for clarity). In order to avoid the computation (see Eq (16)) of a non-physical tip-EC speed, when the predicted lumen size is very low (*R* < 2 *μ*m), a maximum elongation speed, *v*_v-max_, is enforced (see S4 Table). Currently the sprout tip speed does not depend on the MMP concentration or gradient.

Finally, the extension length of a vessel sprout is computed by multiplying the tip velocity with the adopted time increment, Δ*t*_v_, of the vascular network update module (see Solution strategy: Coupled multiscale solver subsection), thus, giving the direction vector of a sprout which is expressed by:

$$d=\left(v_{\text{v}}\;\Delta t_{\text{v}}\right)\;\hat{e}\;.$$ (17)

To reflect numerical and physiological constraints, conditions are placed on capillary growth. If a vascular segment intersects with the external domain boundary, no further extension or branching can take place on this node. Furthermore, given that capillary elongation depends on the local concentration of chemical factors that regulate angiogenesis, a sprout is allowed to grow only if the normalised TAF concentration: *τ* ≥ *τ*^⋆^, with *τ*^⋆^ an imposed threshold value. Branching and anastomosis is modelled by following several rules when running the vascular network module. In anastomosis, if a vascular tip node is within a certain distance range of another vessel (i.e., 40 *μ*m) then anastomosis occurs and a new vascular segment is inserted in the tree to connect the two vascular nodes. In branching, we adopt a similar technique to that proposed by Edgar and colleagues [45], where the formation of branches is modelled as a stochastic process. More precisely, during the simulation and for each angiogenesis time-step, a random number between 0 and 1 is assigned to each node of the vascular network. Then, for each vascular node, a branching probability density function, P, is invoked to predict the likelihood for a vascular node to form sprout. Thus, if the random number falls within the branching probability range then a branch is generated [45]. Function P is equal to the superposition of two normal distribution functions: *(i)* a probability density function of the age of a vascular node, $P_{\text{A}}$, and *(ii)* a probability density function of the relative position of a vascular node with respect to the nearest branching node(s) of the residing vessel, $P_{\text{D}}$. In this work, both probability density functions are also expressed with respect to the local haemodynamics of a vascular segment. The branching parameters of the probability density functions (median and standard deviation) are provided in the second set of parameters in S4 Table.

Non-perfused or hypo-perfused tumour vessels—as opposed to sufficiently or well-perfused ones—are distinguished in the branching algorithm, where the former tumour vessels are allowed to bifurcate more easy when compared to the well-perfused vessels. A detailed description of these processes is given in the following paragraph. The angiogenesis model implicitly promotes the formation of vascular shunts and hence a heterogeneously perfused vascular tree, which has been experimentally observed in tumour vasculature [46]. As such, the angiogenesis model is more likely to generate anastomoses in the far-field of the ECM, whereas the presence of dead-end vessels is pronounced near the tumour–host interface.

#### Capillary wall remodelling

During angiogenesis, blood vessels undergo a remodelling phase which is governed by various biophysical factors. In this section a model that describes the dynamic biomechanical response of the newly formed blood vessels is presented, where simple but rather intuitive constitutive relations of capillary wall remodelling are proposed.

It has been demonstrated that, theoretically, the structural properties of newly formed capillaries can change dynamically in response to various mechanical stimuli [47]. Here the blood vessel wall adaptivity (e.g. capillary radius, wall thickness and pore size) is described by a single variable *t*_*m*_, referred to as the remodelling time. A vessel is defined as fully remodelled when it makes the transition from a relatively poor blood perfusion state into a sufficient blood perfusion state, where the perfusion state is defined through the WSS magnitude, *τ*_f_, (see Eq (9)). Vessel wall remodelling time is set to vary with respect to WSS—induced by the flow of erythrocytes and plasma in the capillaries—via the following expression

tm(τf)={tm-T; if τf≥τ¯ftm-T+Δtm exp[1−(1−τf2τ¯f2)−1]; elsewhere. (18)

Here Δ*t*_m_ = *t*_m-0_ − *t*_m-T_, where *t*_m-0_ and *t*_m-T_ are the corresponding time values for a vessel to reach an upper bound of remodelling and structural integrity when WSS is zero, *τ*_f_ ≃ 0, and above a certain threshold value, $\tau_{\text{f}}\geq {\bar{\tau }}_{\text{f}}$, respectively. In this work, *t*_m-0_ is set to a very high value (= 100 d) to reflect the rather slow process of the vessel wall remodelling, while *t*_m-T_ is chosen empirically to be 10 days. As such, the rate of remodelling of the endothelial wall is described as inversely proportional to the WSS magnitude distribution on the capillary lumen, such that *t*_m-T_ ≪ *t*_m-0_. If we differentiate hypo-perfused from fully functional, well-perfused transport vessels when the mean velocity of the blood flow is below 0.1 mm/s [48], then we can estimate a threshold value of the WSS: ${\bar{\tau }}_{\text{f}}=2.4\times 10^{-8}/R\;$ mm-Hg, which for a capillary of typical size (e.g. *R* = 60 *μ*m) gives ${\bar{\tau }}_{\text{f}}=4\times 10^{-4}$ mm-Hg. Therefore, the microvascular local haemodynamics is related to the remodelling of the capillary lumen (i.e., endothelial cells’ proliferation and basement membrane formation) via the smooth-function constitutive Eq (18). In principle, the remodelling parameters *t*_m-0_ and *t*_m-T_ are selected such that the model predictions are physiologically relevant, and in good agreement with biological experiments. From the results of the biomimetic three-dimensional culture system reported by Seano et al. [49], we take nascent (non-perfused) tumour vessels to form initially tubular structures of very small lumen size of about 5 *μ*m in radius approximately; whereas when tumour vessels transit to a well-perfused state—due to the WSS stimulus—the capillary lumen expands up to 50 *μ*m in radius approximately [22].

In contrast to previous published models in the field [40, 47], only the WSS distribution in a vascular segment is allowed to stimulate capillary wall remodelling here; for example, the model does not incorporate the affects of the microvascular pressure distribution or the metabolic haematocrit-related stimulus. The reasons for this decision are as follows:

1. All inlets and outlets of the vascular tree are designed to serve as arterial and venous ends of the vascular system. However, simulations performed in this analysis (see for example in S12 and S13 Videos) indicate that the vascular pressure, *p*_vsc_ does not change abruptly when anastomosis occurs, vascular shunts are formed, or even when vessels are collapsing during tumour development. It was therefore concluded that *p*_vsc_ has a low effect on capillary wall adaptivity.
2. It was reasoned that fine tuning the extra parameters associated with the elaborate adaptivity model of Pries et al. [50] requires experimental data that are not currently available, hence making the validation of this model less feasible.

The change of the capillary radius is modelled as a function of time and a parameter, *t*_m_, which describes the average time for a blood vessel to become fully remodelled, i.e. when the lumen size and the wall thickness reaches a natural upper ceiling. The capillary radius is expressed in time using the following growth expression

$$R\left(\bar{t}\right)=\left\{\begin{array}{cc} \;R_{\text{min}}\;;\; & \text{if}\;\text{tip}\;\text{vascular}\;\text{node}\\ \;R_{\text{min}}+A_{\text{R}}\;\text{exp}\left[-B_{\text{R}}\;\text{exp}\left[-C_{\text{R}}\;\bar{t}\right]\right]\;;\; & \text{elsewhere} \end{array}\right.\;,$$ (19)

where *A*_R_ = *R*_max_ − *R*_min_, *B*_R_ = 11 and *C*_R_ = 4.4. Furthermore, $\bar{t}=\left(t-t_{\text{g}}\right)/t_{\text{m}}$ is the normalised dimensionless time-variable, *t*_g_ is the actual time when a vascular point is generated, and *t*_m_ the time required for the vessel wall to become fully remodelled, i.e. form a well-structured endothelium with few pronounced fenestrations. The values of the minimum and maximum radius of a newly-formed capillary vessel (*R*_min_, *R*_max_) are provided in S4 Table. It is important to mention here that the pre-existing vascular segments (*t*_g_ = 0) do not undergo any remodelling; as such, their properties are maintained throughout the tumour–angiogenesis simulation unless they become compressed, as discussed at the end of this section. Furthermore, even though the radius of the tumour capillaries can vary considerably and reach a large size, we define a maximum radius to be used in Eq 19 based on experimental data [22, 51].

Under physiological conditions, the wall thickness is assumed to increase linearly as a function of blood vessel remodelling $h\left(\bar{t}\right)=h^{\text{max}}+\left(1-\bar{t}\right)\;h^{\text{min}}$, where *h*^max^ and *h*^min^ are the upper and lower limit of the capillary wall thickness (see S4 Table). A similar approach is taken to modelling vessel pore size, with the radius of the pores, *r*_p_, expected to reduce in time due effects such as vessel wall and basal layer reinforcement and the recruitment of pericytes, according to

$$r_{\text{p}}\left(\bar{t}\right)=\left\{\begin{array}{cc} \;r_{\text{p}}^{\text{min}}\;;\; & \forall \bar{t}\geq 1\\ \;A_{\text{r}}\;{\bar{t}}^{3}+B_{\text{r}}\;{\bar{t}}^{2}+C_{\text{r}}\;;\; & \text{elsewhere} \end{array}\right.\;,$$ (20)

where the coefficients $C_{\text{r}}=r_{\text{p}}^{\text{max}}$, $B_{\text{r}}=3\left(r_{\text{p}}^{\text{min}}-r_{\text{p}}^{\text{max}}\right)$, $A_{\text{r}}=2\left(r_{\text{p}}^{\text{max}}-r_{\text{p}}^{\text{min}}\right)$ are expressed with respect to the maximum and minimum pore size $r_{\text{p}}^{\text{max}}$ and $r_{\text{p}}^{\text{min}}$, respectively.

In summary, when a new vascular node is introduced in the vascular network—as part of a tip-EC elongating or after the formation of a sprouting branch—the corresponding lumen size, wall thickness and pore-size at this node is initialised to *R*_min_, *h*_min_ and *r*_max_ respectively (see parameters values in S3 Table). Evidently, during the remodelling of the capillary wall, when $\bar{t}\geq 1$ (i.e. in time *t* ≥ *t*_g_ + *t*_m_) then the lumen size, wall thickness and pore-size is set *R*_max_, *h*_max_ and *r*_min_ respectively. However, in the event of elevated mechanical forces from the extravascular space the vascular wall may deform, hence, leading to a reduction of the effective lumen size. Detailed explanation of the biomechanics of the wall—with respect to external mechanical loads—is provided in the subsection that follows.

In case a vascular anastomosis occurs, then the formation of the vascular shunt may lead to significant changes of the local haemodynamics at the vascular nodes under consideration, e.g. give rise to high pressure gradients. Subsequently, this will fuel substantial increase of the WSS magnitude. This in turn will speed-up the dilation of the vascular lumen and the capillary wall remodelling, as it is expressed in the above mathematical expressions.

#### Interaction between the tumour–host biomechanics and the capillaries

In a healthy tissue environment, the interstitial fluid pressure remains at low levels (due to the fully functional work of the lymphatic system) and, if physiological in-vivo loading conditions are ignored, residual stresses in the extracellular space can be assumed negligible. However, during abnormal tissue growth—such as in cancer development—compressive mechanical forces, *p*_h_, increase locally (*p*_h_↑) due to the displacement of tissue by the growing cancer mass, while the increased permeability of leaky tumour vessels give rise to the interstitial fluid pressure (*p*_int_↑). To reflect these processes, the model defines a pressure ratio, $\bar{p}$

$$\bar{p}=\left(p_{\text{int}}+p_{\text{h}}\right)/p_{\text{vsc}}\;,$$ (21)

which measures the balance of forces applied both internally and externally to the vessel wall. Here the convention of positive hydrostatic pressure under compression and negative under extension is adopted. Blood pressure at a vascular node, *p*_vsc_, is obtained directly from the fluid flow model, while the solid mechanics pressure (i.e. tr(**S**)/3) is evaluated at the quadrature points of each finite element of the tissue solid domain and then projected at the vascular node, whereas *p*_int_ is averaged from the proximal—to a vascular node—interstitial nodes (see Fig 1C).

The vascular wall is treated as a shell structure under uniform internal (*p*_vsc_) and external (*p*_int_ + *p*_h_) pressure, which can buckle if the overall external load is greater than a critical value. Hence two regimes are defined: if the numerator in Eq (21) is smaller or equal to the denominator then the micro-vessel is under physiological conditions, otherwise the vessel is under compression and the effective lumen radius decreases non-linearly. This work defines a threshold for pressure induced vessel collapse, *p*_c_, such that if $\bar{p}\geq p_{\text{c}}$ then the vessel wall radial deformation increases drastically and the vessel is assumed non-functional (see Fig 2B). In this situation blood flow is blocked while the corresponding vascular segment is “cleaved” from the network; consequently, further capillary sprouting and branching is halted.

In the pressure range $1<\bar{p}<p_{\text{c}}$, the constitutive function relating pressure and radial strain, *e*_r_, is given by: $\bar{p}=1+\left(2p_{\text{c}}-2\right)\left(e_{\text{r}}/e_{\text{c}}\right)-\left(p_{\text{c}}-1\right){\left(e_{\text{r}}/e_{\text{c}}\right)}^{2}$, where *e*_c_ is the critical strain at the point of collapse. As such, the maximum modulus of rigidity of the pressurised vessel wall (when $\bar{p}=1$) reads: *E*_w-max_ = 2(*p*_c_ − 1)/*e*_c_, which in turn is assumed here to vary linearly with respect to the level of “remodelling” of the vascular wall, and by extension the lumen size, the wall thickness and the pore size of the blood vessel. Values of the dimensionless stiffness parameter *E*_w-max_ are provided in S3 Table, where the minimum value corresponds to the mechanical load resistance capacity of the newly formed capillaries having radius *R*_min_ and the maximum to the pre-existing parent vessels of the vascular network. A similar non-linear constitutive biomechanical response was predicted recently by Mpekris et al. [52] where the vessel wall was modelled as a Neo-Hookean material. Here a simple quadratic polynomial constitutive expression is proposed that can reproduce the mechanics of the capillary wall during angiogenesis by encapsulating the effect of interstitial fluid pressure, tissue solid stresses and microvascular pressure.

Having evaluated the radial deformation of the capillary node, *e*_r_, for a given pressure load the corresponding radial stretch ratio can be computed via: λ_r_ = *e*_r_ + 1. If $1<\bar{p}<p_{\text{c}}$ then λ_r_ ≠ 1, therefore, the effective capillary radius scales down via: *R* ← *R*/λ_r_. When $\bar{p}\geq p_{\text{c}}$, then *e*_r_ ≥ *e*_c_ and the scaling parameter is set equal to a very large number (e.g. λ_r_ = 10^3^), in order to describe the collapse the capillary wall and the resulting drastic reduction of the effective lumen size. Evidently, if $\bar{p}\leq 1$ then capillary radius is given by Eq (19) while the wall thickness obeys the remodelling expression provided in the previous subsection.

### Initial and boundary conditions

Using Fig 1A as reference, the solid solver boundary conditions are defined as follows: traction-free (**S** ⋅ **n** = 0, where **n** is the outward surface normal) on the outer surfaces without inlets or outlets, Γ^V^; zero displacements (**u** = **0**) on the outer surfaces with inlets or outlets, Γ^T^ (to avoid rigid body motion); continuity of stress and displacement on the tumour–host interface, Γ^I^. No residual stresses are considered in the present analysis, hence the initial tissue deformation is zero (**F**_e_ = **F**_g_ = **I**).

In order to replicate an initially healthy host environment (i.e. prior to angiogenic tumour development), a uniform distribution of capillaries having approximately uniform diameter, thickness and pore size is imposed initially (see S3 Table), whereas the tumour environment is initially avascular with the mean inter-capillary distance being 0.6 millimetres approximately. The assumption of initial-state parallel, unbranched vessels is based on previous pertinent work in tumour-induced angiogenesis [13, 15].

For the fluid solver, 0.1 mm-Hg interstitial fluid pressure was assigned on the tissue boundary Γ^V^ according to [53], and the interstitial fluid flux was assumed continuous at the tumour–host interface. The vascular pressure, *p*_vsc_, is prescribed at the inlet and outlet vascular nodes of the initial network as 25 mm-Hg and 10 mm-Hg, respectively [54]. Furthermore, throughout the analysis, *p*_vsc_ = 0 is enforced at every node belonging to a collapsed vessel, in order to effectively model the obstruction of the natural flow of erythrocytes and plasma at that part of the vascular network.

For the biochemical solver, zero-flux boundary conditions of all species are applied at the outer boundary of the tissue region, Γ (and note the domain is chosen to be sufficiently large that this does not impact the solution in the main body of the tissue). Continuity of concentration and its flux are assumed at the tumour–host interface. The initial conditions are *τ* = *ξ* = *μ* = 0 everywhere except for the healthy tissue domain, where *ξ* = 1. The initial condition for the ordinary differential Eq (1) describing the ECM density is *ϵ* = 1 in the entire region of the analysis domain.

### Solution strategy: Coupled multiscale solver

**Fig 3:**
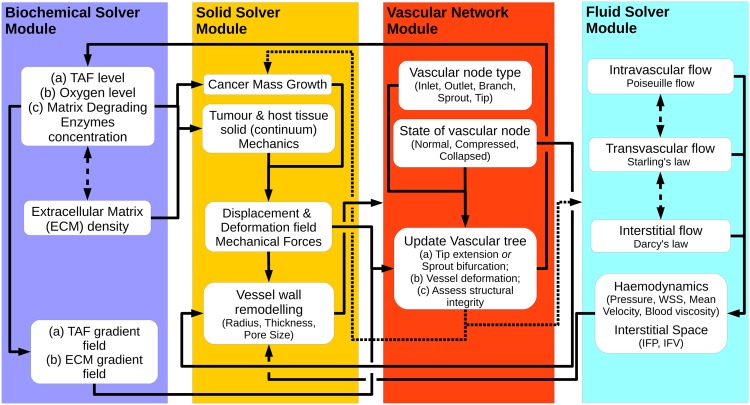
Flow diagram of the coupled multiscale solver. Schematic representation of the work flow diagram of the three-dimensional tumour growth and angiogenesis model that shows the interaction between the biochemical module, the vascular network module and the solid and fluid mechanics solver modules.

The problem under consideration is transient by nature and the modelling framework, as described in the previous section, consists of four interconnected core components, namely the *Vascular Network Module*, the *Biochemical Solver Module*, the *Solid Solver Module* and the *Fluid Solver Module*. Fig 3 illustrates the building blocks of the proposed modelling framework in a flow diagram and the interaction amongst them. The numerical procedure of the coupled tumour-growth and tumour-induced angiogenesis multiscale solver is outlined in the following section.

The mathematical model identifies three different time-discretisation scales, one for each of the first three solver modules:

1. The time integration step of the reaction-diffusion equations in the Tissue biochemical model subsection.
2. The time step between successive solutions of the linear momentum equation for the tissue solid mechanics.
3. The time increment (see Eq (17)) used to update the vascular network.

In order to ensure that the explicit time integration scheme of the biochemical equations produces stable solutions, the Courant-Friedrichs-Lewy condition needs to be satisfied; given the smallest-element size in the 3D mesh, a very short time step has been adopted of the order of a few seconds. Similarly, to ensure that the nonlinear solid mechanics solver converges after a reasonable number of Newton-Raphson iterations (e.g. four or five), the corresponding time increment has been set equal to approximately one hour. Finally, after parametrically investigating the results produced by the vascular network update module, time increment Δ*t*_v_ has been set to six hours. It is important to note here that using large values of Δ*t*_v_ can generate long, straight vascular segments, hence influencing the prediction of the vascular tree “shape” and organisation; whereas using very small Δ*t*_v_ can increase the computational cost substantially, since the generation of very small vascular segments will result in a rapid increase in the number of degrees-of-freedom in the final system of the fluid mechanics solver. In light of the model variables *ρ*_v_ and *ρ*_t_, to ensure that a newly-formed vascular segments passes through (and not crosses) any tissue element (see Fig 1), the 3D mesh density of the analysis domain must be properly selected, as follows. The maximum elongation length from Eq (17) gives: (*v*_v-max_ Δ*t*_v_) = 0.0625 mm (see material parameter S4 Table). Therefore, the minimum element size of the 3D mesh must be greater or equal to maximum elongation length—in the present analysis the minimum edge length (at the centre of the analysis domain; see S1 Fig) is 0.07 mm approximately.

The algorithmic structure is as follows:

**Initialisation**:
  Load the 3D finite element grid of the analysed domain. Label elements as either tumour or healthy tissue and assign corresponding material properties (see tables in the Supporting Information). Initialise deformations and stresses at the quadrature points, and set initial nodal values for all state variables (see Tissue biochemical model subsection). Load the 1D grid of the vascular network and associate each vascular node with a 3D tissue element of the analysis domain. The initial vascular tree comprises of (parent) capillaries of the host tissue, having geometric properties defined in S4 Table.
**Iterate in Time**:
  1. **Run the Biochemical Solver**:
    Numerically solve the parabolic Eqs (12)–(14) together with Eq (1), and compute state variables: {*τ*, *ξ*, *μ*, *ϵ*} at the vertex nodes of the 3D FE mesh. Compute the gradients of the species at the quadrature points of the grid using Lagrange interpolation. Pass these variables to the *Solid Solver Module* to compute tumour growth and update the structural integrity of the ECM; also inform the *Vascular Network Module* accordingly in order to inform blood-vessel sprouting and development according to Eq (15).
  2. **Run the Solid Solver**:
    Numerically solve the nonlinear elliptic equations for the solid domain to evaluate nodal displacements [55]; then compute the deformation, **F**, and stress field, **S**, at the quadrature points of the finite elements. Pass these variables to the *Vascular Network Module* to evaluate the blood vessels’ sprouting orientation vectors, and predict the distensibility of the vascular network due to tumour–host tissue deformation.
  3. **Update the Vascular Network**:
    Evaluate the extension at every node of the 1D vascular network using Eqs (15) and (16). Loop for all vascular nodes of the tree, and check if the node has $\bar{p}\geq p_{\text{c}}$: if this is true then check if the node can sprout or extend its tip (*τ* ≥ *τ*^⋆^) and evaluate the geometric features (radius/thickness/pore-size) of the existing and newly formed capillaries; otherwise ignore subsequent calculations for this vascular node. Also, mark connected nodes as collapsed (due to local obstruction of flow) unless anastomosis has occurred. Compute and update the density of functional vascular segments, *ρ*_v_, for each 3D finite element. Project the solution (i.e. displacements, mechanical stresses) from the *Solid Solver Module* onto the vascular network nodes. Finally, assess the state (i.e. normal/compressed/collapsed) of the vascular segments in the tree.
  4. **Run the Fluid Solver**:
    Solve Eqs (4)–(6) to compute the interstitial fluid pressure in the extravascular space nodes, and the blood pressure distribution at the vascular tree nodes. Evaluate the transvascular pressure difference and the interstitial fluid velocity.
  5. **Re-update the Vascular Network**:
    Re-assess the state of the vascular segments inside or proximal to the tumour region, and revise the list of collapsed blood vessels and update the microvascular pressure and interstitial fluid pressure distribution.

The above procedure is repeated until the termination of tumour growth. Details about the FE implementation of the proposed tumour-induced angiogenesis and growth model are provided in the S1 File.

## Results and Discussion

A series of simulations are performed and parameters related to the growth of the tumour, the evolution of intra-tumoural fluid and solid stresses, as well as the vascular density and functionality are presented and validated against published experimental data. The predicted vascular geometry is compared with geometrical characteristics of experimentally-derived vascular networks that we calculated from angiography images of MCaIV murine mammary adenocarcinomas. We find that the model is able to replicate features of angiogenic tumour growth in MCaIV mouse tumours, and also demonstrate that a mechano-sensitive model is necessary to provide a representative physiological description. Details of the analysis performed to obtain the mouse model data are provided in S2 File.

For the simulations, a cubic domain (12 mm edge length) of tissue is considered, with an initial cancer mass embedded in the cube centre, represented by a sphere of 1 mm diameter. This tumour geometry is representative of tumours grown in immunodeficient mice.

A three-dimensional finite element mesh is constructed using Gmsh, consisting of 3320 hexahedra and 3963 nodes. S1 Fig depicts the modelling domain, including the finite element meshes, the tumour location and size, and the initial network of capillaries embedded in the ECM. Note that the tumour is initially avascular.

The material parameters adopted in this study are listed in the Supporting Information Tables. Most parameters are taken from the literature, with some chosen specifically from murine mammary adenocarcinoma experiments. First of all we discuss the validation of the model, and next we present the simulation results.

### Model validation from the literature

Here we present the validation of the model in terms of vascular and interstitial fluid pressure and velocity, and vascular density predictions against published data.

**Fig 4:**
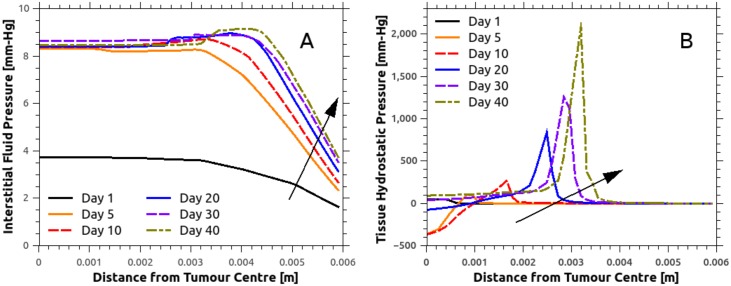
Spatial distribution of interstitial fluid pressure (IFP) and the tissue hydrostatic pressure (THP). Arrows show the direction of time increasing. A: IFP remains relatively flat within the cancer mass, while the predicted value agrees very well with reported in-vivo data on MCaIV murine mammary carcinomas [57]. Pronounced tumour growth results in an increase in the IFP in the peri-tumoural area, up to approximately 1.5 mm-Hg. B: THP peaks at the tumour periphery, symptomatic of increased compressive solid stresses due to the cancer mass growth in this region. Increased circumferential solid stresses at the tumour periphery induces compression of the vessels, which are subsequently pruned. Note that negative THP denotes tension and positive compression.

**Fig 5:**
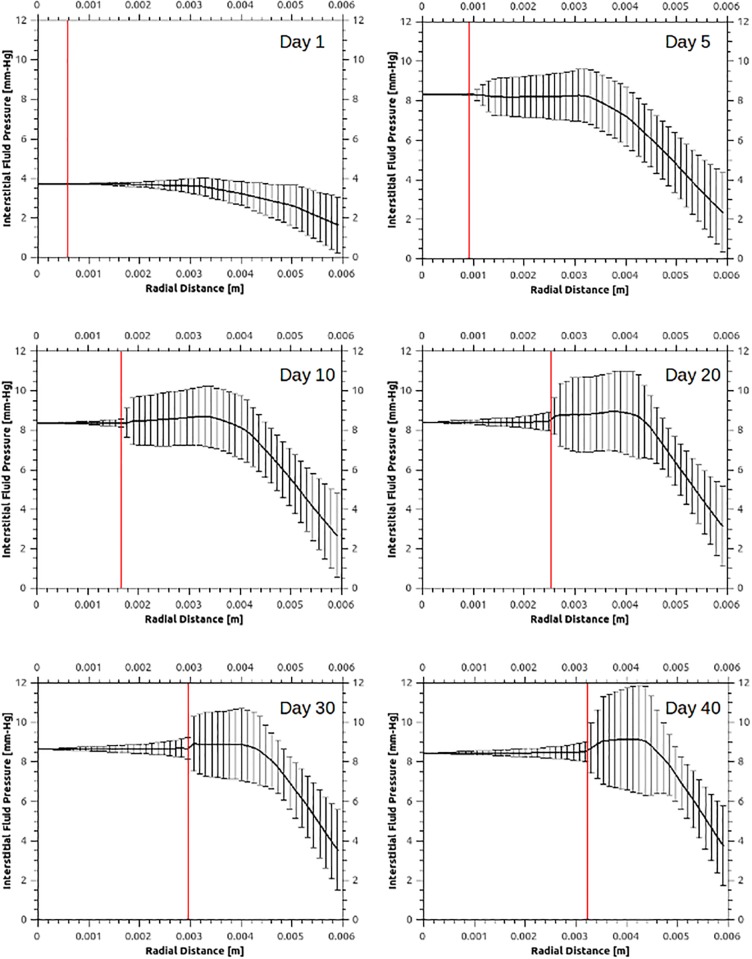
Interstitial fluid pressure (IFP) spatial distribution at various time instants. Within 24 hours of relatively slow avascular tumour growth, tumour-secreted angiogenic chemical factors have sufficiently diffused in the extracellular space. In the tumour-induced angiogenesis simulation, day-1 marks the formation and elongation of new blood vessel sprouts. The vertical red line in the plots defines the tumour–host interface boundary which corresponds to the averaged tumour radius, where the centre of the cancer mass is at zero radial distance. The solid line corresponds to the mean IFP distribution, evaluated at fourteen azimuthal directions, while the vertical bars denote the IFP standard deviation. These plots also illustrate the gradual increase of the cancer mass, while depicting the significant variability of IFP in the vicinity of the tumour interstitium.

To qualitatively validate the fluid solver predictions, Fig 4A presents the average interstitial fluid pressure, *p*_int_, distribution in the tumour and the peri-tumoural area for various time instants, where the centre of the tumour is at zero radial distance. Complementary to this plot, the significant variability of the IFP is shown in Fig 5 as a function of radial distance from the tumour centre for a number of time points. Interestingly, a rapid increase in the IFP is observed within one day, as a result of the formation and elongation of new and hyper-permeable capillary sprouts. This reflects the patho-physiological nature of such sprouts, which typically have a discontinuous endothelial lining and no basal membrane, rendering them hyper-permeable. Boucher and colleagues [56] measured the IFP for two rat tissue tumour types (mammary adenocarcinoma and Walker 256 carcinoma) using micro-pipettes. Comparing the numerical predictions, shown in Fig 4A, with the experimental results reported by Boucher et al. (see Fig 3 in [56]), a strong qualitative agreement is observed in the drop of the IFP away from the tumour–host interface. The numerical predictions also show that the IFP reaches a maximum plateau throughout the tumour, which, despite the significant increase of tumour volume—from 19.06 mm^3^ in day-10 to 141.37 mm^3^ in day-40—remains relatively stable. The mean plateau IFP predicted by the model is approximately 8.3 mm-Hg. This falls within the experimentally measured pressure range reported previously for tissue-isolated (9.1±3.9 mm-Hg; see Table 2 in [56]) and subcutaneous (7.8±3.8 mm-Hg; see Table 2 in [56]) small-size tumours (<1 g), while it is also in qualitative agreement with the IFP measurements [37] for MCaIV-type murine mammary carcinomas (5.6±1.2 mm-Hg; see Fig 3F in [37]).

Further qualitative validation of the fluid solver was performed by examining the interstitial fluid velocity (IFV), calculated from Darcy’s law (see Eq (11)). Assuming that the tissue hydraulic conductivity is constant, the IFV is driven only by gradients of the IFP local to the tumour–host tissue. However, as illustrated in Fig 5, the IFP remains relatively flat in the tumour region and decreases rapidly when moving from the tumour to the healthy tissue. Therefore, the IFV in the tumour centre is negligible compared to the corresponding pronounced IFV measured near the tumour boundary. This effect is visualised in S10 and S11 Videos: the IFV gradually increases in the tumour centre during angiogenesis, whereas abrupt IFV elevation is observed at the tumour periphery. The latter observation is made more evident in S12 and S13 Videos, where the IFV peak values are predicted primarily in the vicinity of collapsing blood vessels of the original network. Thus, drastic changes in the functionality of the tumour vasculature lead to loss of balance in the microvascular pressure distribution and the intra- and extravasation flux of plasma/proteins in the capillaries. This drives dynamic changes in the IFP, which subsequently result in larger IFP gradients and hence a larger IFV. As such, the above predictions confirm the hypothesis that there is significant interstitial hypertension in the tumour. The maximum estimated value of the fluid velocity in the interstitium was approximately 0.15 *μ*m/s, which is in qualitative agreement with the experimentally measured values (0.6±0.2 *μ*m/s) reported in the early paper of Chary and Jain [58].

**Fig 6:**
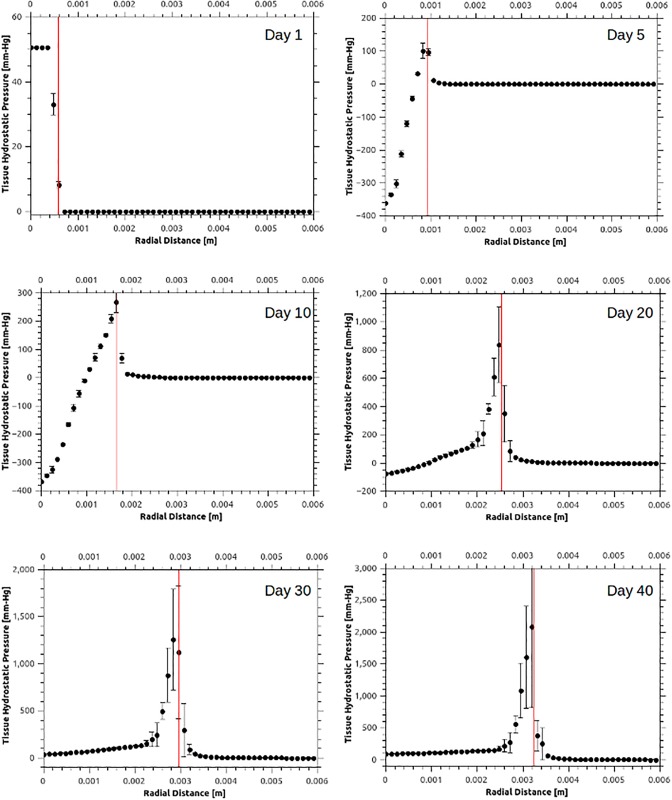
Average tissue hydrostatic pressure (THP) spatial distribution at various time instants. Various snapshots of THP—i.e. the mean solid stress—distribution evaluated at fourteen azimuthal directions, plotted with respect to the radial distance from the tumour centre. Positive pressure is compressive and negative extensive, while the vertical bars denote standard deviation. The vertical red line in the plots defines the tumour–host interface boundary, while the vertical bars denote the THP standard deviation. Notably, during the tumour development mechanical forces are propagating, in the form of a pressure wave, with an approximately linearly increasing amplitude in time.

In Fig 4B, the magnitude of the mechanical forces in the stroma—i.e. the tissue hydrostatic pressure (THP) which is equal to one third of the trace of the stress tensor, also referred as the mean solid stress—is shown as a function of the radial distance from the centre of the tumour at several time points. Also Fig 6 reports the spatial distribution of THP at various time instants, where both the average value of the THP and the standard deviation (in error bars) is shown. In contrast to the fluid-pressure distributions in the interstitium shown in the Fig 5, THP shows substantial spatio-temporal variability primarily at the interface of the tumour and the host tissue (vertical red bar in Fig 6). This can be explained by the anisotropic growth driven by the irregular angiogenic vasculature, which provides a non-uniform supply of oxygen to the tumour. These results support the idea of a heterogeneous force environment at the tumour periphery, consistent with the observations reported in the past [4, 59].

**Fig 7:**
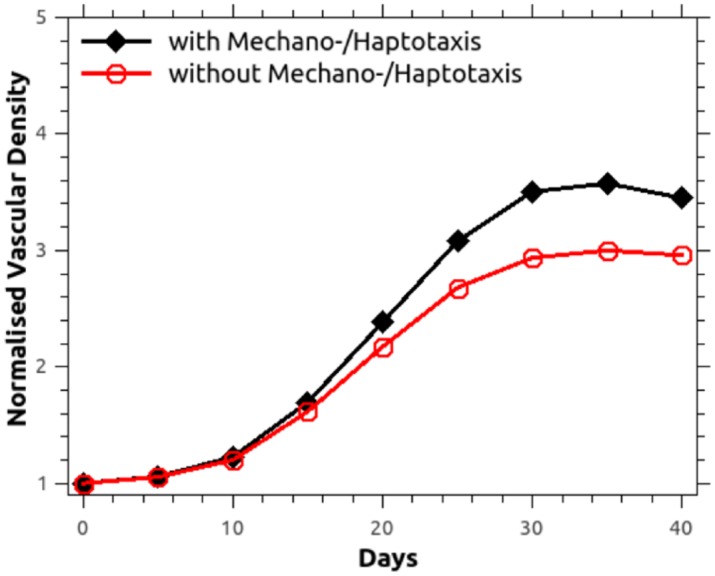
Normalised vascular density when mechano- and haptotaxis is included or discarded in the angiogenesis model. Comparison of the numerically predicted normalised vascular density when blood vessel sprouting is modulated by chemo-, mechano- and haptotaxis against the simplified chemotaxis case. Vascular density is defined as the ratio of the surface area of the blood vessels to the tissue volume, and is normalised against the corresponding initial value (day-0).

Numerous features of our evolving vascular structures recapitulate experimental observations. First of all, the mean inter-capillary distance increased from around 0.557 mm to 0.771 mm at the end of simulation time. This is consistent with experimental measurements, for example in stage IIb and III carcinomas of the cervix (measured using histochemistry, see [60]), where the average inter-capillary distance in the cancerous tissue was around 304±30 *μ*m higher than that in healthy tissue. Secondly, our predictions of tumour vascular density (defined as blood vessel surface area divided by the tissue volume, normalised against the corresponding healthy tissue value) are reported in Fig 7, for mechano- and haptotactic stimuli switched on or off. The normalised vascular density increases in time as the tumour grows, reaching around 3.4 and 3.0 with/without the mechano-/haptotactic stimuli by the end of the simulation. This is in good agreement with reported measurements [61–63], which lie in the range 3.3 to 5.0, with the inclusion of mechanical stimuli improving the agreement with measured observations.

### Validation against new experimental analysis

Vascular density and inter-capillary distance alone do not assess the functionality of the three-dimensional vasculature in its entirety. In particular, solute (such as oxygen and drugs) delivery is determined by diffusion distances from the vessels, and influenced by the spatial architecture and organisation. We consider the averaged power spectrum of the distance map computed on the vascular network, which was originally proposed by Risser et al. [64] in brain tumours (and further used by Baish et al. [65]) for assessing the diffusive capacity of drugs in normal tissue and tumours. We introduce two scaling parameters λ_v_ and *δ*_v-max_, following the approach of Baish [65]; the first measures the shape of the space between blood vessels, whereas the second estimates the shortest distance between a tissue point and vessel.

**Fig 8:**
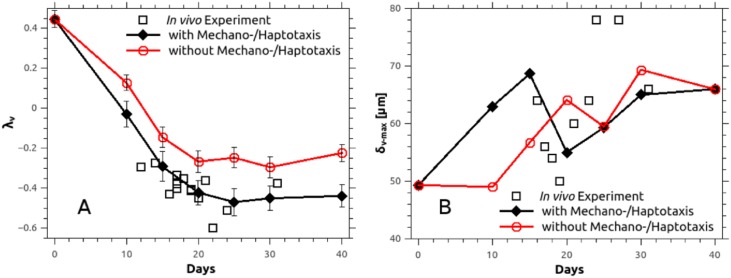
History plots of the parameters characterising the morphology of the microvascular tree. Numerically predicted parameters λ_v_ and *δ*_v-max_ with respect to time, compared to in-vivo measurements in murine mammary carcinoma (MCaIV-type) [22]. A: The geometrical exponent, λ_v_, is obtained after linear regression on the pair of data: frequency of voxels versus the distance to nearest vessel (*δ*_v_) [65] while the vertical bars denote standard deviation of the mean.

We compare here the model predictions of parameters λ_v_ and *δ*_v-max_ against the experimentally measured values, extracted by quantifying the vascular structure in murine breast carcinomas using in-vivo imaging. The images of the tumour vasculature were obtained in a previous research study using the optical frequency domain imaging [22]. However, brief description of the present analysis of the in-vivo images can be found in S2 File. The comparison of the two scaling parameters is summarised in Fig 8. Model predictions of λ_v_ agree very well with the in-vivo data, and this agreement is improved with the mechano-/haptotactic stimuli switched on compared to off. This is an extremely promising validation step of our in-silico cancer model, which provides strong evidence that our inclusion of mechano- and haptotaxis is highly relevant to predicting and testing delivery of diffusible agents to vascular tumours. The analysis of the parameter *δ*_v-max_ is less conclusive. The trend offered by the experimental data points here is less well-defined (see Fig 8B); our model certainly predicts differing behaviours with the mechano- and haptotactic terms switched on/off, and there are currently insufficient experimental data points to draw a conclusion on which model prediction is correct.

### Oxygen-driven growth and network remodelling

**Fig 9:**
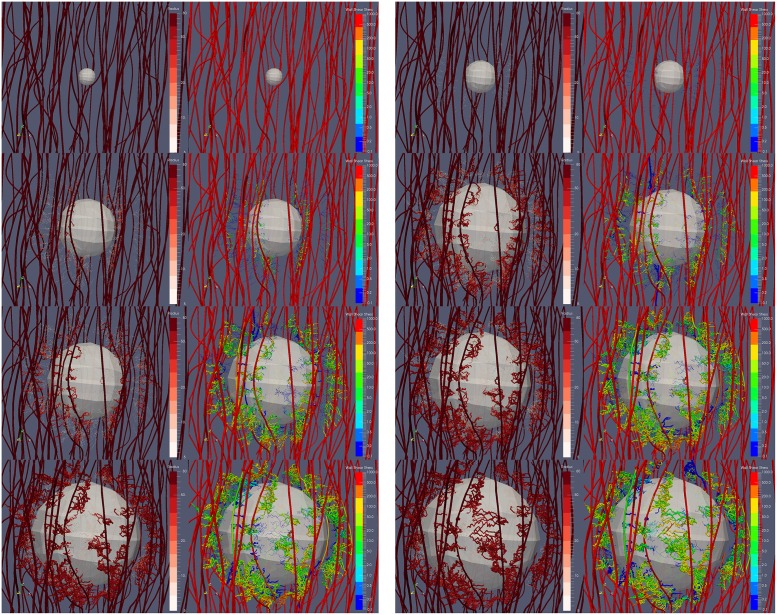
Snapshots of the tumour growth and angiogenesis simulation illustrating the remodelling of the microvasculature and haemodynamics. Visualisation of the developing tumour and the formation of new vascular sprouts, branches and anastomoses over a period of 40 days (from top to bottom and from left to right: day-0, day-5, day-10, day-15, day-20, day-25, day-30 and day-40). In the contour maps, the vascular lumen radius range is 5–80 *μ*m and the wall shear stress magnitude range is 10^−1^—10^3^ mm-Hg (on logarithmic scale).

Non-isotropic growth is driven by the non-uniform spatial distribution of capillaries at the tumour periphery and hence—as described by Eq (3) and the relation defining **F**_g_—the resulting oxygen distribution. In Fig 9 (see also S1 video), the tumour is illustrated to grow due to the oxygen transcending from the vasculature and diffusing in the interstitial space. The visualisation also demonstrates dynamic network remodelling: not only do the vessels lack a regular hierarchy and structure, but they also display a higher degree of tortuosity than the initial (healthy) vascular network. In S2 Fig, the numerically predicted increase of the cancer mass as a function of time (in days) is illustrated.

### Model enables quantification of vessel perfusion

**Fig 10:**
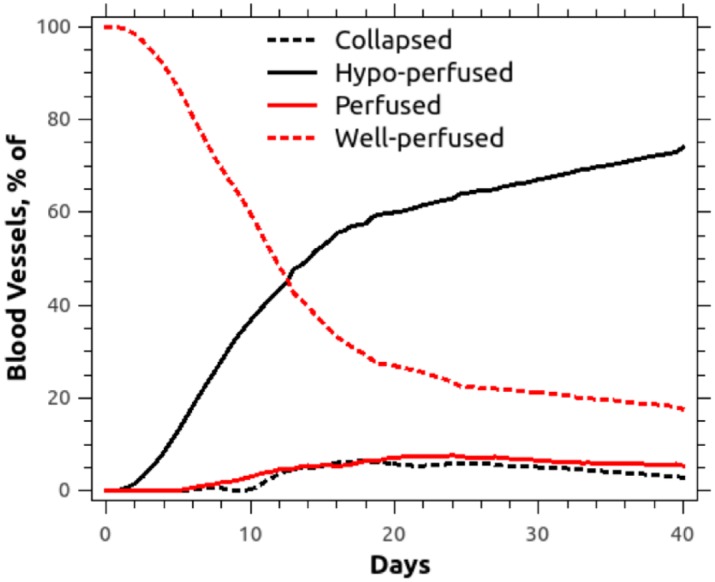
Perfusion state of the vascular network in time. Following [48], hypo-perfused vessels are characterised as those with mean intravascular velocity below 0.1 mm/s, perfused vessels are those falling in the range of [0.1, 0.5] mm/s, and the rest (>0.5 mm/s) are considered well-perfused blood vessels. The percentage of collapsed vessels fell between approximately 4–6% with respect to the total amount of vessels in the vascular tree.

The in-silico cancer model enables a quantitative investigation of blood flow in the evolving vascular network. S5 fig compares the mean blood flow velocity with vessel diameter (considering functional i.e. non-collapsed vessels only) at different time points. Such information is extremely hard to measure accurately using experimental methods, given the requirement to image flow in individual microvessels of micron-level diameters; a validated in-silico framework is therefore highly valuable in providing insight into microvessel functional behaviours. Fig 10 shows the perfusion state of the vasculature as a function of time. The blood vessels are distinguished into functional and non-functional (i.e. collapsed) vessels, with the former being categorised according to their mean blood velocity (MBV) as hypo-perfused (BMV <0.1 mm/s), perfused (BMV in the range 0.1–0.5 mm/s) and well-perfused (BMV >0.5 mm/s) (this follows the convention proposed by Kamun et al. [48]). The proportion of hypo-perfused vessels increases quickly over the first 10 to 15 days, primarily driven by the unregulated vessel sprouting. After this time, the increase slows as a consequence of anastomosis and branching of vessels.

### Mechano- and haptotaxis play an important role in tumour-induced angiogenesis

To date, mathematical models of angiogenesis have mostly focused on chemotactic (e.g. VEGF) and haptotactic tip cell migration as determinants of vascular sprouting and branching (key example papers [18, 23, 66–68]). These studies do not incorporate the impact of mechanotaxis in determining vascular topology, and both vascular and tumour growth. Here we investigate the hypothesis that vascular structure during tumour growth is influenced by chemo-, mechano- and haptotactic stimuli in combination. Specifically, we carry out simulations when chemotaxis alone determines the vessel tip extension rate and direction, and compare them against those where all three stimuli are included (see Eq (15)).

In Fig 7 we compare the normalised vascular density (as defined previously) as a function of time with chemotaxis in isolation, with the case of combined chemo-, hapto- and mechanotaxis. When only chemotaxis is active, the pathway of the tumour vessels is dictated by the gradients of TAF, where TAF—owing to the isotropic diffusion of these chemical cues in the ECM—is distributed spherically. Therefore, the elongation direction vector of the newly-formed sprouts points towards the tumour centre. However, owing to the growing tumour mass, solid stresses increase dramatically at the peritumoural stroma (especially at day 10 and onwards; see Fig 6), inducing collapse of the infiltrating nascent vessels when mechanical cues for vessel growth are included. Therefore, when chemo-, hapto- and mechanotaxis are combined, the pattern of angiogenesis changes significantly. Careful inspection of the simulation results reveals that the elevated mechanical forces at the peritumoural stroma re-direct the vessels to elongate circumferentially and, thus, increase the likelihood for the formation of anastomoses and vascular shunts. Notably, anastomoses are evident after day 10 owing to the growing population of microvessels and the significant increase of branches adjacent to the tumour. Summarising the above, and as shown in Fig 7, our in-silico model predicts an increase of the normalised vascular density in the combined taxis case of angiogenesis.

Also, Fig 8 compares the numerically predicted scalar parameters λ_v_ and *δ*_v-max_ in the two cases of taxis. The combined mode model produces higher normalised vascular densities, more consistent with experimental measurements, and also provides an excellent prediction of λ_v_, as discussed in the previous section. This analysis indicates that hapto- and mechanotaxis may play an important role in determining the density and three-dimensional spatial arrangement of angiogenic vessels in tumours; in turn, these structural features of the vasculature are key in predicting diffusion of solutes (e.g. oxygen, drugs) into the interstitial space, and thus drug penetration and efficacy. Our model incorporates these mechanical stimuli, and could be used in the future to optimise tumour drug delivery and dosage. Furthermore, the model has predictive capability to characterise tumour solid stresses, and their interplay with tumour growth.

**Fig 11:**
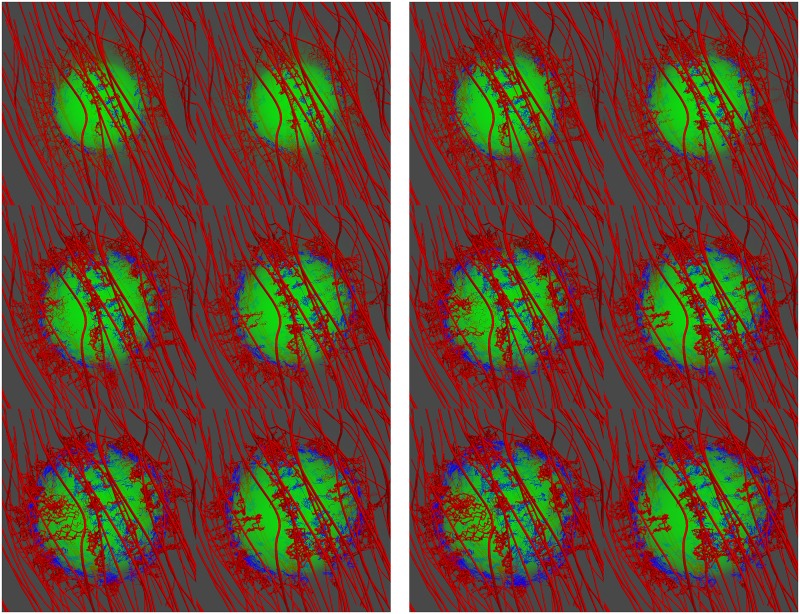
Comparison snapshots showing the effect of taxis to the tumour angiogenesis model predictions. Tumour induced angiogenesis simulation results are compared when chemo-, mechano- and haptotaxis is taken into account for the capillary sprouting kinematics (left image) and when chemotaxis applies only (right image), while each row corresponds (from left to right and from top to bottom) in days 15, 20, 25, 30, 35 and 40. Functional blood vessels are coloured red while collapsed vessels are in blue. The green-colour cloud denotes the TAF concentration level in the extracellular matrix, where both the host-tissue and tumour domain is invisible for illustration purposes.

Fig 11 illustrates in snapshots the in-silico predictions of tumour-induced angiogenesis when chemo-, mechano- and haptotaxis is taken into account (images on the left of each column), and when chemotaxis applies only. Notably, from day 30 onwards, the tumour vessels follow a rather radial extension pattern in the pure chemotaxis case. However, when mechano- and haptotactic cues are also considered, the growing vessels are observed to encapsulate (rather than penetrate) the tumour. This “tumour framing” effect becomes more striking when the minimum TAF threshold required for angiogenesis, τ*, is lowered (see following subsection). Also, particularly at days 35 and 40, the formation of vascular shunts is also more pronounced when all three taxes (referred to as the ‘combined mode’) are included, vessel tortuosity is increased, and the presence of anastomoses is also more frequent (see also S14 Video). These are characteristic features of tumour vasculature, and it is highly promising that they are promoted by an in-silico model that includes mechanotaxis.

### Angiogenesis is strongly determined by TAF-levels and vascular-wall rigidity

**Fig 12:**
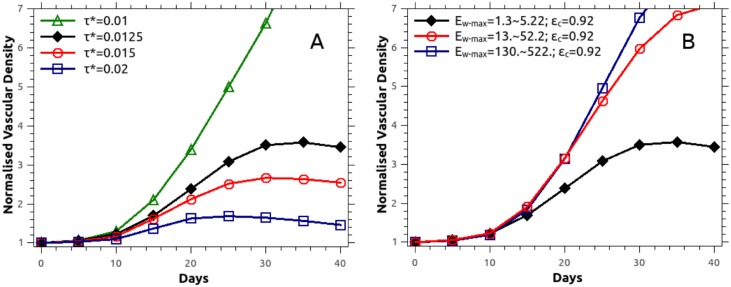
Predictions of the normalised vascular density as a function of time. Increase of tumour vascular network density with respect to time when varying: A: the TAF angiogenesis threshold τ*, and B: the capillary wall stiffness, *E*_w-max_. See also description in Fig 7 for the definition of the normalised vascular density.

Finally, the dependency of angiogenic vascular growth on various model parameters was examined. A sensitivity analysis of all parameters was performed and those with the largest influence on the normalised vascular density were identified, namely the TAF concentration and vascular wall stiffness. Fig 12A shows the normalised vascular density as a function of time for various values of the TAF threshold, (τ*), above which angiogenesis is permitted (all other parameters were kept constant). A non-linear dependency of the vascular density on τ* is observed, with a value of τ* = 0.02 producing a monotonic increase. This reflects the inherent non-linearity of TAF-induced vessel production, and suggests that the TAF threshold can quash other limiting factors—such as collapse from solid stress—which cause a decrease in vascular density above day 35 for the lower threshold values. This establishes TAF as a dominant factor in angiogenesis, which is in agreement with previous findings [69].

To test the response on vascular mechanics, Fig 12B shows the normalised vascular density as a function of time for various values of the maximum wall stiffness, *E*_w-max_; all other parameters were kept constant. Specifically, in the baseline test 1.3 ≤ *E*_w-max_ ≤ 5.22, which—as described in the previous section, and given that the critical radial strain a capillary can sustain is approximately 0.92—is equivalent to the critical pressure for capillary wall collapse 1.6 ≤ *p*_c_ ≤ 3.4. In the first sensitivity test the stiffness was increased by a factor of 10, and in the second test by a further factor of 10. This produced a highly nonlinear response, with a monotonic increase in the vascular density in time observed for both sensitivity tests. Considering Eq (21), a capillary segment is assumed collapsed when the sum of the interstitial fluid pressure and the tissue hydrostatic pressure is at least *p*_c_-times larger than the microvascular pressure at this segment. As such, these results suggest that increasing *p*_c_ by a factor of 10 renders the vessels essentially immune to collapse by external solid and fluid pressure.

**Fig 13:**
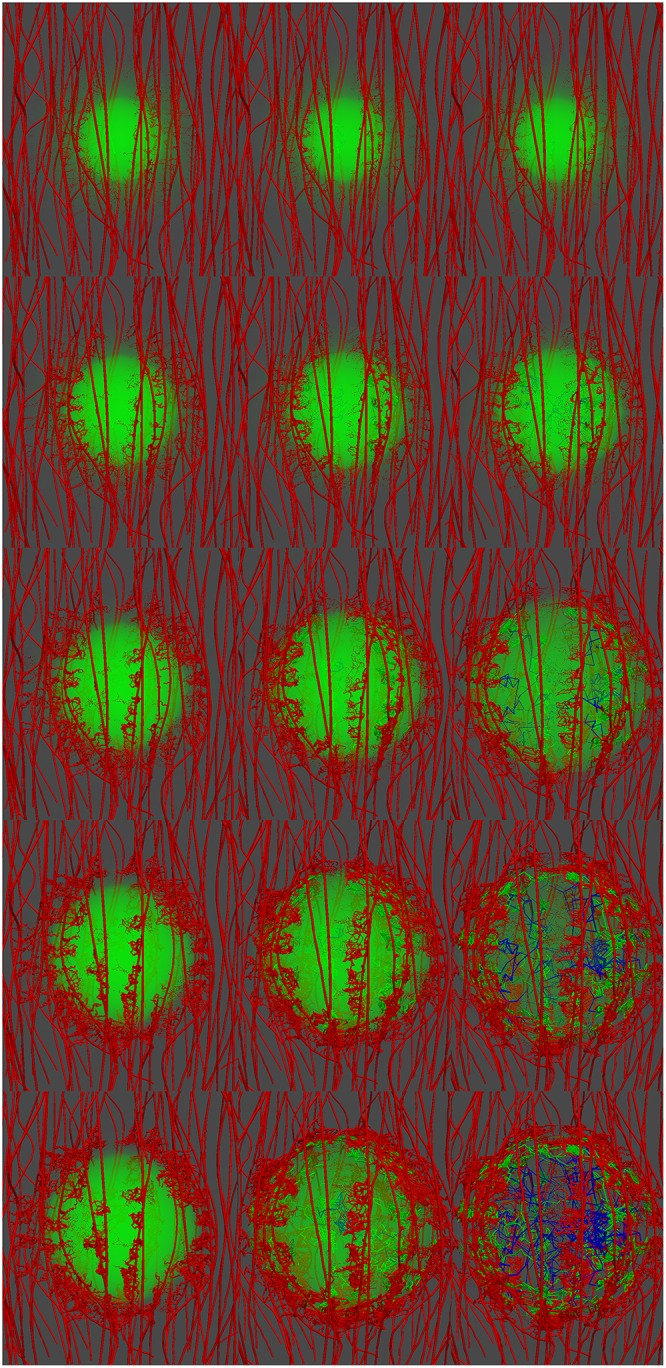
Comparison snapshots showing the effect of vascular wall stiffness to the tumour angiogenesis model predictions. Microvascular wall stiffness (from left to right) takes values: *E*_w-max_ = 1.3–5.22, *E*_w-max_ = 13.—52.2 and *E*_w-max_ = 130.—522. respectively, while each row corresponds (from top to bottom) to day-15, day-20, day-25 and day-30. Blood vessels are coloured green if they reside at the peri-tumoural area, red if they are located in the rest of the healthy tissue domain, and blue if the are inside the tumour. Note that for lower values of stiffness, *E*_w-max_, nascent vessels are non-existent inside the tumour as opposed to higher values. The green-colour cloud denotes the TAF concentration level in the extracellular matrix, where both the host-tissue and tumour domain is invisible for illustration purposes.

Fig 13 illustrates the impact of the microvascular wall stiffness in the predictions of the tumour growth and angiogenesis simulator (see also the animation of S15 Video). It is evident from this figure that enabling vessel wall more resilient (rightmost column of images), nascent vessels can resist and withstand the elevated mechanical forces at the tumour periphery. Thus, they can penetrate the tumour whose growth is speeded up and is slightly pronounced—provided that functional and well-perfused vessels supply the core of the cancer mass with vital nutrients and oxygen—as opposed to for example the baseline case (leftmost column of images). In summary, varying the wall stiffness in our coupled model produces a similar effect on the vascular density predictions to that when varying the TAF threshold triggering angiogenesis.

### Conclusion

This work presents a validated three-dimensional mathematical and computational framework that encompasses solid tumour growth and tumour induced angiogenesis. The in-silico cancer model has been implemented in our in-house, open-source numerical platform FEB3 (see for details S1 file). The proposed multiscale approach is capable of modelling the mechanical interactions between healthy and cancerous tissues, and associated vasculature, with the solid (tissue) and the fluid (blood) part of the tumour environment modelled separately. The important novel contributions of our model are: (i) the dynamic remodelling of the vascular network under mechanical stress during tumour growth, (ii) the incorporation of mechanotaxis (alongside chemo- and haptotaxis) in the determination of vessel sprouting orientation and speed, (iii) the collapse of tumour blood vessels as a consequence of solid stress produced by the surrounding tissue. The model recapitulates experimental observations of fluid and solid pressure (and fluid velocity) distributions, vascular density and three-dimensional spatial arrangement, with the improvement between experimentally measured and theoretically predicted vascular measurements increasing upon the inclusion of hapto- and mechano-tactic (alongside chemotactic) stimuli in the model. This supports our hypothesis that hapto- and mechanotaxis play an important role in determining the density and 3D spatial arrangement of tumour induced vasculature. As further data that quantify tumour vascular structures become available, we will continue to test the applicability of the metrics *δ*_v-max_, λ_*v*_ in capturing intra-tumour heterogeneity, as well as tumour-to-tumour variations, alongside the ability of our mechanotactic model in predicting these changes.

Despite the complexity of the proposed framework, our model remains subject to some limitations; particularly, we do not model space- and time-varying haematocrit and relative blood viscosity [50, 54, 70], or the impact of the lymphatic system. The focus of this study was the investigation of the coupling between tumour vasculature, growth and the generation of solid stresses, and therefore these features were ignored at this stage, for the sake of simplicity. Also, we envisage to incorporate in a future version of the tumour angiogenesis module an elaborate model of vascular wall-remodelling and biomechanics. For example, the biomechanical properties of the microvascular wall (i.e. lumen size, thickness and pore size) could explicitly be described with respect to local gradients of chemical cues affecting the endothelium integrity (i.e. stiffness, permeability). As such, we will model the spatio-temporal biomechanics of the microvasculature in a more physiologically realistic manner; this will, for example, overcome the simplification of isotropic mechanical compression. In addition, we plan to extend the present in-silico tumour model to account for polyclonal cell populations and, thus, investigate the importance of the angiogenesis in the interplay between different cancer cell phenotypes (i.e. proliferative versus invasive phenotype) under different vascularisation regimes [71], as well for various tumour-related cell types (e.g. cancer-associated fibroblasts, cancer-associated macrophages, etc.).

However, the increased sophistication of our computational framework results in a large number of model parameters presented in S1–S4 Tables. Most of the model parameters were determined independently from the others based on previous studies and experimental data. Other parameters—not found in the literature—were defined in the current study so that model predictions to be physiologically relevant. The good agreement of model predictions with experiments that has been demonstrated in this work validates the choice of model parameters. Furthermore, we performed a parametric analysis of the parameters that play a key role in the angiogenesis procedure and in the compression of the blood vessels (i.e. mechano-/haptotaxis, TAF threshold for angiogenesis, vessel wall stiffness), which are the two factors of tumour progression that our work has been focused on. Variation of other model parameters is expected to change the results only quantitatively, while qualitatively the model predictions and the conclusions of this study will remain the same. We also note that the model framework we present is deterministic; a natural next step would be to investigate variability in the model predictions induced by stochastic features, for example, of the initial vasculature (length, diameter and separation of vessels), informed by distributions of these data as measured in practice. We leave this to future work, but note its important in understanding tissue heterogeneity, both within an individual tumour, and between tumours of the same/different type.

Finally, our model can be further used as a platform for the study of the delivery of drugs to solid tumours by adding equations for the transport of the therapeutic agent into the vascular network, across the vessel walls and into the tumour interstitial space [4, 26, 72]. Additionally, the efficacy of strategies that target the tumour microenvironment to enhance the delivery of drugs—such as vascular normalization and stress alleviation treatments—can be studied in more detail than by existing models.

## Supporting Information

S1 File FE implementation of the tumour angiogenesis and growth model. (PDF)

S2 File Quantification of the structure of in-vivo tumour vasculature. (PDF)

S1 Fig Three-dimensional finite element mesh and vascular network. A: Clipped mesh, showing the internal structure of the grid. B: The extracted tumour region (shown as a small sphere) with the complete micro-vasculature rendered as red tubes.
(TIFF)

S2 FigTumour volume and approximate diameter development in time.Increase of tumour volume, Ω^T^, and approximate diameter ($\sqrt[{3}]{4\Omega^{\text{T}}/3\pi }$) with respect to time (in days). In all simulations the tumour is allowed to grow approximately seven times in diameter. The plots do not retain one-to-one correspondence due to the non-isotropic growth of the cancer mass.(TIFF)

S3 Fig History plots of the interstitial fluid pressure (IFP) and the tissue hydrostatic pressure (THP) at the centre of the cancer mass. A: IFP grows exponentially as a result of the rapid production of immature, leaky vessels. The sharp drops and fluctuations of the IFP coincide with the pruning of neighbourhood blood vessels (due to growth-induced stresses) and the eventual collapse of some parent capillaries that supply the extravascular space with blood. However, interstitial tension is restored by the generation of new sprouts and subsequent anastomoses that continuously fuel the leaky vessels with more extravasating biological fluid. B: The solid-phase pressure (referred here as THP) at the tumour centre increases initially towards significant tension. This can be justified by the passive biomechanical response of the hypo-perfused cancer core, whose cellular status is rather dormant. Subsequently, after day-8, the core of the tumour transits into a necrotic state, while getting compressed by the highly proliferative tumour periphery.
(TIFF)

S4 Fig Histograms illustrating the dynamic changes of the vascular network (lumen diameter versus length) at various time instants. The normalised vascular length on the vertical axis is determined by the fraction of the total length of vascular segments of a specific diameter size over the total length of the functional (non-collapsed) vessels at each time frame. The labels on the horizontal axis correspond to the following blood vessel diameter range: (1) functional vessels of diameter 4–10 *μ*m, (2) functional vessels of diameter 10–20 *μ*m, (3) 20–30 *μ*m, etc. The histograms highlight the dilated capillaries in the tumour-associated vascular network, which confirms established in-vivo observations in solid tumours [37]. The corresponding normalised length of the collapsed vessels (not plotted below) is computed: 0.043, 0.186, 0.391, 0.623, 0.929, 1.278 and 1.639 for days 10, 15, 20, 25, 30, 35 and 40 respectively. Comparing the figures with the above data, it becomes evident that this is due to the progressive compression and pruning of blood vessels throughout the analysis.
(TIFF)

S5 Fig Histograms of the intravascular velocity with respect to the lumen diameter of the functional capillaries in the vascular tree. Intravascular velocity is given in mm/s, while the labels on the horizontal axis correspond to the following diameter range: (1) 4–10 *μ*m, (2) 10–20 *μ*m, (3) 20–30 *μ*m, etc. Note that bars exceeding 1 mm/s blood mean velocity (not shown here) are observed to have an exponential increase trend with respect to vessel diameter from day-30 to day-40, while from day-15 to day-25 the distribution is rather random.
(TIFF)

S1 Video Tumour growth and angiogenesis animation that illustrates the capillary radius dynamics. Visualisation of the tumour growth and angiogenesis model predictions, over a period of 40 days, where the tumour is shown as a transparent object and the blood vessels are rendered as tubes, thus, depicting the adaptive behaviour to capillaries to the mechanical stimuli (i.e. fluid viscous forces and solid stresses). The colour contour on the vascular network represents the vessel radius, in *μ*m. Since vessel wall remodelling is modulated by the local flow WSS magnitude, note the direct correlation of the radius development with respect to the WSS in S3 Video. Note also that some parent vessels (originally shown white) are compressed due to the local increase of the mechanical forces, while others during the course of the analysis are collapsed and hence vanish from the network. Finally, note the formation of vascular shunts at the top left and inferior of the tumour, and the pronounced tortuosity of the newly formed vessels in the the peri-tumoural area.
(MP4)

S2 Video Tumour growth and angiogenesis animation that illustrates the intravascular blood mean velocity (BMV). As in S1 video, this animation illustrates BMV, in mm/s. Here it is clearly observed that non-functional (i.e. collapsed) vessels are pruned from the vascular network throughout the analysis, as explained in the Capillary wall remodelling subsection. Note that the mean velocity of the well perfused vessels (shown in dark red) can reach up to several mm/s, while functional poorly perfused vessels are shown white. In this regard, it is interesting to observe that a few of the parent vessels have also collapsed, and hence obstruct the natural blood flow.
(MP4)

S3 Video Tumour growth and angiogenesis animation that illustrates the wall shear stress (WSS) magnitude. This video shows dynamic changes of the WSS magnitude, in mm-Hg, induced by the blood as it flows through the vessels, over a period of 40 days. As expected, poorly perfused vessels have low WSS, whereas the highly perfused parent vessels have high WSS.
(MP4)

S4 Video Tumour growth and angiogenesis animation that illustrates the tumour-angiogenic growth factor (TAF) distribution and the functional state of the vascular network. This video shows TAF spatial distribution as a green cloud—secreted by the tumour cells—while diffusing into the extracellular space of the host tissue (c.f. Eq (12)). The functional blood vessels are rendered as red tubes, while the collapsed vessels appear as thin blue lines.
(MP4)

S5 Video Animation of the blood pressure distribution in the vascular network during a 40-day tumour-induced angiogenesis process. Inlet and outlet vascular nodes are randomly chosen, where a 25 mm-Hg arterial and a 10 mm-Hg venous pressure is applied respectively. Intravascular blood pressure distribution, also referred to as the capillary hydrostatic pressure, is given in mm-Hg.
(MP4)

S6 Video Three-dimensional contour plot of the interstitial fluid pressure (IFP) distribution in the tumour-host soft tissue domain and the blood vessels. The growing tumour is shown as a transparent sphere, while the vascular network is presented as a scaffold by showing only the centreline tree. The predicted IFP solution, in mm-Hg, is projected from the nodes of the three-dimensional finite element grid onto the vascular nodes via a conventional interpolation scheme (i.e. the inverse distance algorithm).
(MP4)

S7 Video Opposite view of S6 Video. See caption of supplementary material S6 Video.
(MP4)

S8 Video Dynamic three-dimensional contour plot of the tissue hydrostatic pressure (THP) distribution in the tumour–host tissue domain and the blood vessels. For an interpretation of the tumour and vascular network geometry consult the description of S6 video, while THP is given in mm-Hg.
(MP4)

S9 Video Opposite view of S8 Video. See caption of supplementary material S8 Video.
(MP4)

S10 Video Three-dimensional contour plot of the interstitial fluid velocity (IFV) distribution in the tumour-host soft tissue domain. The blood vessels are coloured with respect to the microvascular (blood) pressure distribution. The growing tumour is shown as a transparent sphere, while the vascular network is presented as a scaffold by showing only the centreline tree. Note also the logarithmic scale used for displaying the contour of the IFV, in *μ*m/s.
(MP4)

S11 Video Opposite view of S10 Video. See caption of supplementary material S10 Video.
(MP4)

S12 Video Animation of the microvascular pressure and interstitial fluid velocity. Combined visualisation, as in S10 Video, of the microvascular pressure and the IFV using vectors, during a 40-day period of tumour growth. The transparent growing sphere corresponds to the tumour, with the centre-line of the vascular tree shown only. IFV increases smoothly within the tumour during the simulation, as a result of the space-varying IFP as shown in Fig 5. However, IFV peaks at some points, mainly in the vicinity of collapsing blood vessels of the original network. This is due to sudden pruning of highly perfused vessels that leads to the loss microvascular pressure balance, which dynamically changes the intravasation and extravasation of plasma/proteins in the vessels. This, subsequently, dramatically modifies the distribution of the IFP.
(MP4)

S13 Video Opposite view of S12 Video. See caption of supplementary material S12 Video.
(MP4)

S14 Video The effect of taxis to the tumour angiogenesis model predictions. This animation compares (left hand side) the tumour growth angiogenesis simulation result when chemo-, mechano- and haptotaxis is taken into account for the capillary sprouting kinematics—as described in Eq (15)—with (right hand side) the result when only chemotaxis is considered. Similar to S4 video, the green cloud corresponds to the distribution of TAF in the ECM, while the functional blood vessels are rendered as red tubes and collapsed as blue. It is interesting to observe on the left the tangential distribution of vessels at the tumour periphery, as opposed to the relatively radial direction of vessels on the right hand side simulation. The former leads to the formation of vascular shunts in the peri-tumoural stroma, while blood vessels are more likely to anastomose and hence enhance their perfusion.
(MP4)

S15 Video The effect of vascular wall stiffness to the tumour angiogenesis model predictions. In this video we provide a direct comparison of the simulation results, in a 30-day window, for various values of the capillary wall stiffness. As illustrated in the legend of Fig 12B, the simulation cases from left to right correspond to *E*_w-max_ = 1.3–5.22 (baseline value range), *E*_w-max_ = 13.—52.2, and *E*_w-max_ = 130.—522. respectively. Blood vessels are coloured green if they reside at the peri-tumoural area, red if they are located in the rest of the healthy tissue domain, and blue if the are inside the tumour. Again, the green cloud in the centre of each visualisation denotes the concentration of TAF secreted by the tumour cells. It is interesting to see the excessive vessel pruning in the baseline case compared to the pronounced vascularisation of the rightmost simulation. Note also in the latter case the significant speed up of tumour growth due to the presence of perfused vessels in the core of the cancer mass.
(MP4)

S1 Table Solid mechanics model parameters. List of model parameters associated with the *Solid Solver Module*. Set of parameters in the last three rows are non-applicable for the host tissue region.
(PDF)

S2 Table Fluid mechanics model parameters. List of model parameters associated with the *Fluid Solver Module*. Cells marked with an asterisk denote shared values for both tissue types.
(PDF)

S3 Table Biochemical model parameters. List of model parameters associated with the *Biochemical Solver Module*. Cells marked with an asterisk denote shared values for both tissue types, while “NA” denotes non-applicable.
(PDF)

S4 Table Vascular network model parameters. List of model parameters associated with the *Vascular Network Module*. Parameters with a star (⋆) correspond to non-perfused or hypo-perfused vessels, while those with a dagger (†) correspond to well-perfused vessels. The parameters with a double dagger (‡) denote the pre-set parameter values of the original vascular network.
(PDF)
