# Supplementary material for: A Validated Multiscale In-Silico Model for Mechano-sensitive Tumour Angiogenesis and Growth
Source: PLoS Comput Biol. 2017 Jan 26;13(1):e1005259. doi: 10.1371/journal.pcbi.1005259 (PMC5268362; doi:10.1371/journal.pcbi.1005259)
Supplement: S1 File — (PDF) [file pcbi.1005259.s001.pdf]

SUPPORTING INFORMATION

A Validated Multiscale In-silico Model for Mechano-sensitive Tumour Angiogenesis and Growth

Vasileios Vavourakis, Peter A. Wijeratne, Rebecca Shipley, Marilena Loizidou, Triantafyllos Stylianopoulos, David J. Hawkes

FE implementation of the tumour angiogenesis and growth model

The coupled mechano-biological multiscale finite element procedure has been implemented in a scalable C++ code, and incorporated into the existing in-house numerical analysis framework *FEB3*. *FEB3* has been designed in an object-oriented manner and facilitates parallel computation using the message passing interface technology of the *MPICH* library, while it is founded on the following high-performance, open-source numerical libraries:

- *blitz++* is a meta-template library in C++ which was utilised in *FEB3* for tensor algebra and multi-dimensional tensor manipulation [1].
- *GNU Scientific Library* is an ANSI-C library that contains an wide range of mathematical routines (over 1000 in total) such as random number generators, special functions, statistics, numerical differentiation, data fitting, etc. (see online documentation: `https://www.gnu.org/software/gsl/manual/html_node/`) [2].
- *METIS*[1] and *ParMETIS*[2] is a pair of libraries containing established algorithms for partitioning graphs, partitioning finite element meshes and producing fill-reducing orderings for sparse matrices, in serial and in parallel computing respectively [3].
- *MPICH* is a standardized and portable message-passing system which is a communication protocol for programming parallel computers.
- *PETSc* is a suite of data structures and routines for the solution of scientific applications, and is been used within *FEB3* in solving linear and nonlinear systems [4, 5]. *PETSc* is also integrated with *MPICH* to facilitate parallel computations, while it communicates with *METIS* and *ParMETIS* for sparse system partitioning.
- *libMesh*[3] is the top-level library used by *FEB3*. *libMesh* is an object-oriented C++ framework for the numerical simulation of partial differential equations using arbitrary unstructured discretisations on serial and parallel platforms [6], while it integrates with high-performance computing libraries such as *PETSc*.

FEB3 is freely available upon request from: `https://bitbucket.org/vasvav/feb3-finite-element-bioengineering-in-3d/wiki/Home`.

The three-dimensional FE tissue mesh and the one-dimensional vascular network mesh was decomposed and parallelised across multiple processors using the *ParMETIS*

[1] `http://glaros.dtc.umn.edu/gkhome/metis/metis/overview`
[2] `http://glaros.dtc.umn.edu/gkhome/metis/parmetis/overview`
[3] `http://libmesh.github.io/`

library. All simulations presented in this work were carried out on a desktop machine having an Intel Xeon E5-2620 CPU (2.0 GHz $\times$ 6) and 15.6 GB RAM memory, operating Linux (kernel version: *3.13.0-71-generic*).

References

1. Veldhuizen T. Blitz++ User's Guide: A C++ class library for scientific computing; 2006. Available from: `http://blitz.sourceforge.net/resources/blitz-0.9.pdf`.

2. Galassi M, Davies J, Thailer J, Gough B, Jungman G, Alken P, et al.. GNU Scientific Library Reference Manual; 2013. Available from: `http://www.gnu.org/software/gsl/`.

3. Karypis G, Kumar V. MeTis: Unstructured Graph Partitioning and Sparse Matrix Ordering System, Version 4.0; 2009. `http://www.cs.umn.edu/~metis`.

4. Balay S, Gropp WD, McInnes LC, Smith BF. Efficient Management of Parallelism in Object Oriented Numerical Software Libraries. In: Arge E, Bruaset AM, Langtangen HP, editors. Modern Software Tools in Scientific Computing. Birkhäuser Press; 1997. p. 163–202.

5. Balay S, Abhyankar S, Adams MF, Brown J, Brune P, Buschelman K, et al. PETSc Users Manual. Argonne National Laboratory; 2014. ANL-95/11 - Revision 3.5. Available from: `http://www.mcs.anl.gov/petsc`.

6. Kirk BS, Peterson JW, Stogner RH, Carey GF. `libMesh`: A C++ Library for Parallel Adaptive Mesh Refinement/Coarsening Simulations. Engineering with Computers. 2006;22(3–4):237–254.
