# Supplementary material for: A Validated Multiscale In-Silico Model for Mechano-sensitive Tumour Angiogenesis and Growth
Source: PLoS Comput Biol. 2017 Jan 26;13(1):e1005259. doi: 10.1371/journal.pcbi.1005259 (PMC5268362; doi:10.1371/journal.pcbi.1005259)
Supplement: S2 File — (PDF) [file pcbi.1005259.s002.pdf]

SUPPORTING INFORMATION

A Validated Multiscale In-silico Model for Mechano-sensitive Tumour Angiogenesis and Growth

Vasileios Vavourakis, Peter A. Wijeratne, Rebecca Shipley, Marilena Loizidou, Triantafyllos Stylianopoulos, David J. Hawkes

Quantification of the structure of in-vivo tumour vasculature

Images of the tumour vasculature were obtained in a previous research study by Vakoc et al. [1] using the optical frequency domain imaging (OFDI) technique. The mammary adenocarcinoma MCaIV-cell line was implanted in the dorsal skinfold chamber of severe-combined immunodeficient mice. Tumours were allowed to grow to a size of approximately 5 mm, which is considered sufficient for a well developed tumour vascular network. Subsequently, high resolution 3D images of the entire tumour vasculature were collected using OFDI angiography. Imaging was performed every second day so that at least five imaging sets per tumour were obtained at different time points. To quantify the structure of the tumour vasculature obtained with the OFDI technique or generated by the computer simulations, we used two measures of vascular geometry: the maximum distance in the tissue from the nearest blood vessel, $\delta_{\text{v-max}}$, and a measure of the shape of the spaces between vessels, λ_{v}. In previous research [2] it has been shown that the values of these measures are distinct between normal and tumour vessels. A detailed description of the algorithms used for the derivation of the values of these parameters can be found in the paper of Baish et al. [2]. Briefly, the 3D images were binarised and random walk simulations of one million walkers were carried out. Walkers were released at random voxels in the extravascular space. At each time step, the walkers were allowed to move at random to an adjacent voxel until they reach the wall of a neighbouring blood vessel and the distance between the initial position of the walker and the vessel wall was recorded. Subsequently, the number of walkers was plotted as a function of the distance to the vessels. The maximum distance to the vessels was described by the quantity: $\delta_{\text{v-max}}$, while the parameter λ_{v} was derived from the slope of the plot at the range: $\delta_{\text{v}} < \delta_{\text{v-max}}/3$.

References

1. Vakoc BJ, Lanning RM, Tyrrell JA, Padera TP, Bartlett LA, Stylianopoulos T, et al. Three-dimensional microscopy of the tumor microenvironment in vivo using optical frequency domain imaging. Nature medicine. 2009;15(10):1219–1223.

2. Baish JW, Stylianopoulos T, Lanning RM, Kamoun WS, Fukumura D, Munn LL, et al. Scaling rules for diffusive drug delivery in tumor and normal tissues. Proceedings of the National Academy of Sciences of the United States of America. 2011;108(5):1799–1803.
