# Supplementary figures and images for: A Validated Multiscale In-Silico Model for Mechano-sensitive Tumour Angiogenesis and Growth
Source: PLoS Comput Biol. 2017 Jan 26;13(1):e1005259. doi: 10.1371/journal.pcbi.1005259 (PMC5268362; doi:10.1371/journal.pcbi.1005259)

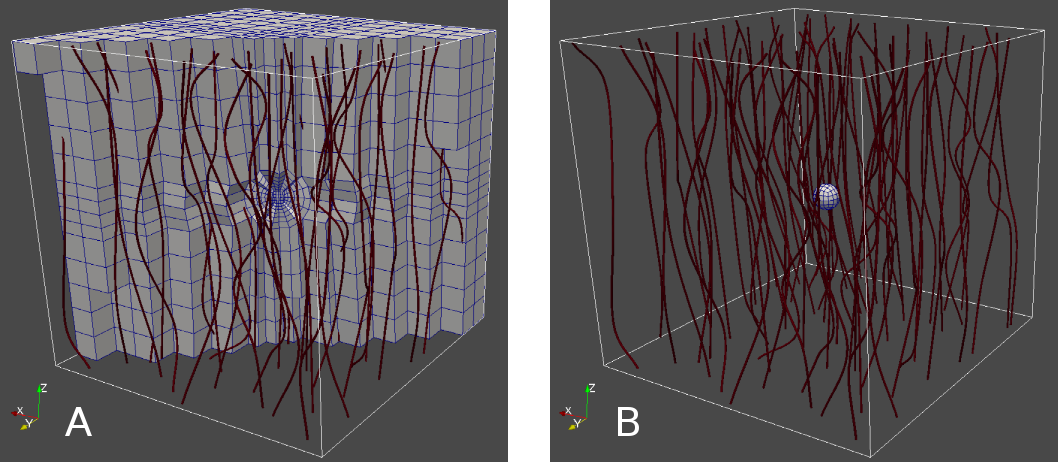

Supplement: S1 Fig — A: Clipped mesh, showing the internal structure of the grid. B: The extracted tumour region (shown as a small sphere) with the complete micro-vasculature rendered as red tubes. (TIFF) [file pcbi.1005259.s003.tiff]

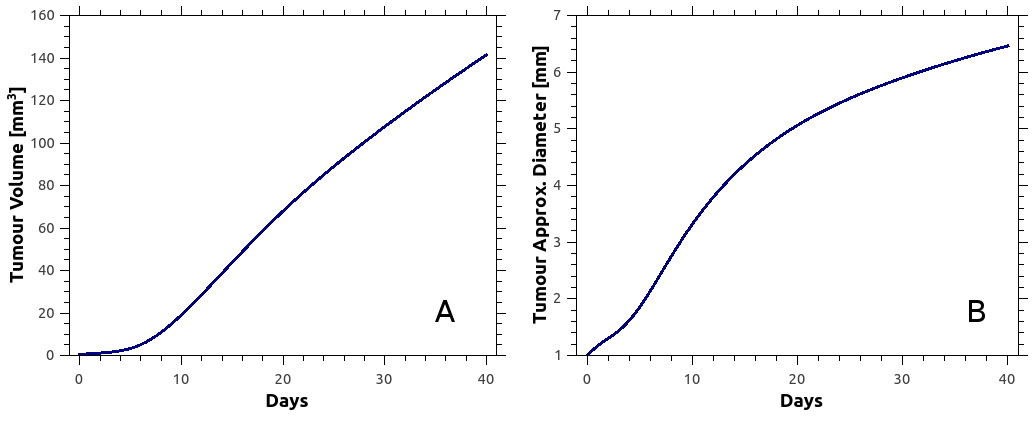

Supplement: S2 Fig — Increase of tumour volume, ΩT, and approximate diameter (4ΩT/3π3) with respect to time (in days). In all simulations the tumour is allowed to grow approximately seven times in diameter. The plots do not retain one-to-one correspondence due to the non-isotropic growth of the cancer mass. (TIFF) [file pcbi.1005259.s004.tiff]

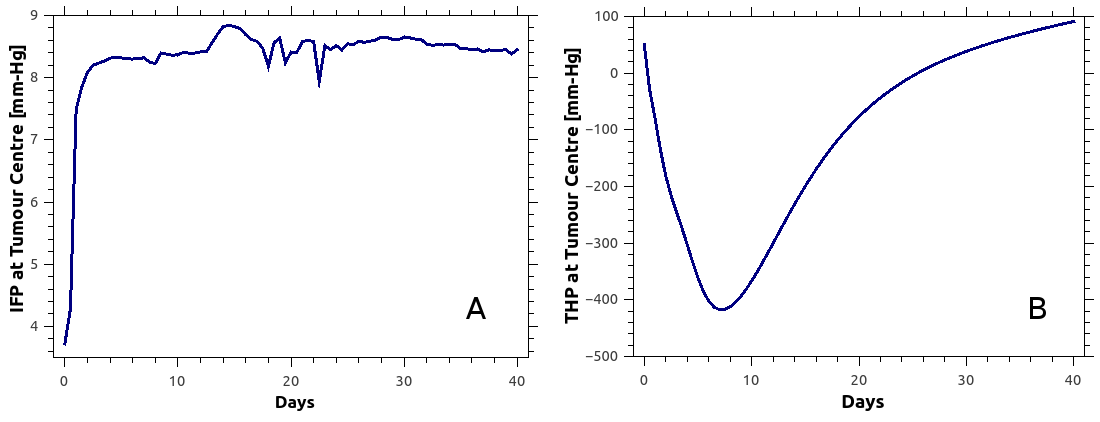

Supplement: S3 Fig — A: IFP grows exponentially as a result of the rapid production of immature, leaky vessels. The sharp drops and fluctuations of the IFP coincide with the pruning of neighbourhood blood vessels (due to growth-induced stresses) and the eventual collapse of some parent capillaries that supply the extravascular space with blood. However, interstitial tension is restored by the generation of new sprouts and subsequent anastomoses that continuously fuel the leaky vessels with more extravasating biological fluid. B: The solid-phase pressure (referred here as THP) at the tumour centre increases initially towards significant tension. This can be justified by the passive biomechanical response of the hypo-perfused cancer core, whose cellular status is rather dormant. Subsequently, after day-8, the core of the tumour transits into a necrotic state, while getting compressed by the highly proliferative tumour periphery. (TIFF) [file pcbi.1005259.s005.tiff]

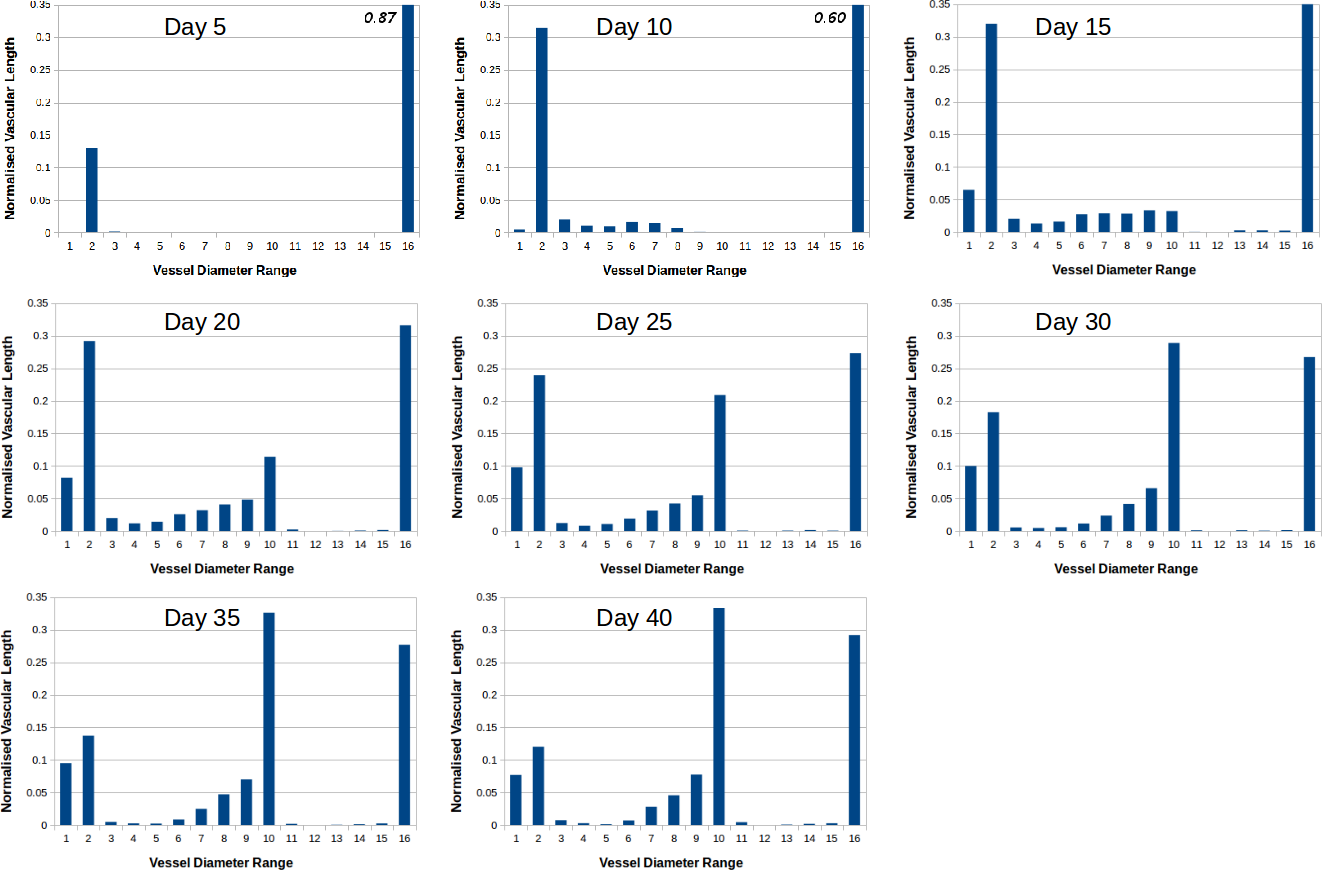

Supplement: S4 Fig — The normalised vascular length on the vertical axis is determined by the fraction of the total length of vascular segments of a specific diameter size over the total length of the functional (non-collapsed) vessels at each time frame. The labels on the horizontal axis correspond to the following blood vessel diameter range: (1) functional vessels of diameter 4–10 μm, (2) functional vessels of diameter 10–20 μm, (3) 20–30 μm, etc. The histograms highlight the dilated capillaries in the tumour-associated vascular network, which confirms established in-vivo observations in solid tumours [37]. The corresponding normalised length of the collapsed vessels (not plotted below) is computed: 0.043, 0.186, 0.391, 0.623, 0.929, 1.278 and 1.639 for days 10, 15, 20, 25, 30, 35 and 40 respectively. Comparing the figures with the above data, it becomes evident that this is due to the progressive compression and pruning of blood vessels throughout the analysis. (TIFF) [file pcbi.1005259.s006.tiff]

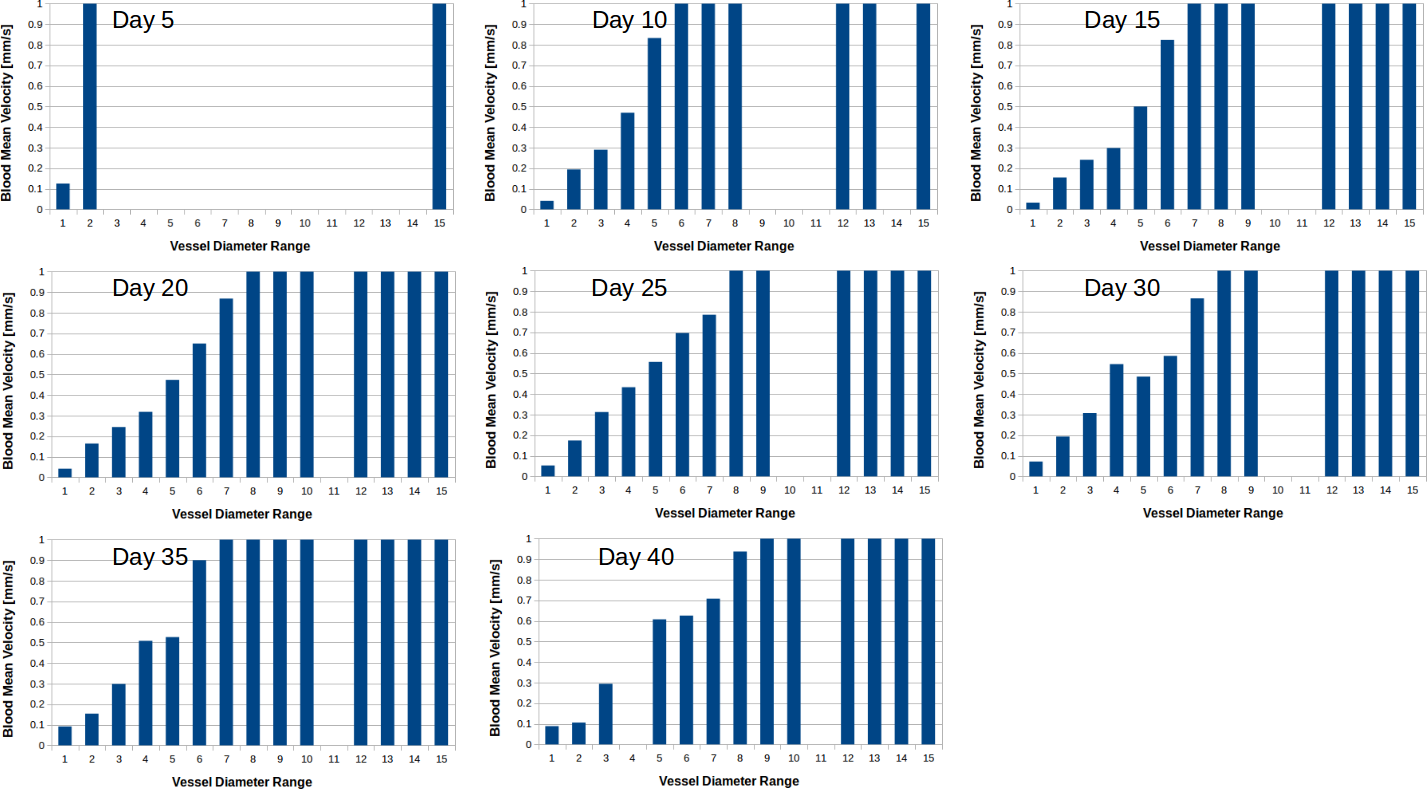

Supplement: S5 Fig — Intravascular velocity is given in mm/s, while the labels on the horizontal axis correspond to the following diameter range: (1) 4–10 μm, (2) 10–20 μm, (3) 20–30 μm, etc. Note that bars exceeding 1 mm/s blood mean velocity (not shown here) are observed to have an exponential increase trend with respect to vessel diameter from day-30 to day-40, while from day-15 to day-25 the distribution is rather random. (TIFF) [file pcbi.1005259.s007.tiff]
