# Supplementary material for: A Validated Multiscale In-Silico Model for Mechano-sensitive Tumour Angiogenesis and Growth
Source: PLoS Comput Biol. 2017 Jan 26;13(1):e1005259. doi: 10.1371/journal.pcbi.1005259 (PMC5268362; doi:10.1371/journal.pcbi.1005259)
Supplement: S1 Table — List of model parameters associated with the Solid Solver Module. Set of parameters in the last three rows are non-applicable for the host tissue region. (PDF) [file pcbi.1005259.s023.pdf]

SUPPORTING INFORMATION

A Validated Multiscale In-silico Model for Mechano-sensitive Tumour Angiogenesis and Growth

Vasileios Vavourakis, Peter A. Wijeratne, Rebecca Shipley, Marilena Loizidou, Triantafyllos Stylianopoulos, David J. Hawkes

Solid mechanics model parameters

List of model parameters associated with the *Solid Solver Module* (see Fig 3). Set of parameters in the last three rows are non-applicable for the host tissue region.

**Parameter**	**Description**	**Host**	**Tumour**	**Source**
κ [kPa]	Mooney-Rivlin model	23.34	50.4	[1,2]
c_{10} [kPa]	Mooney-Rivlin model	5.	18.9	[1,2]
c_{20}, c_{01}, c_{02}, c_{11} [Pa]	Mooney-Rivlin model	0.	0.	[1,2]
$\alpha_{\rm g}$, $\beta_{\rm g}$, $\gamma_{\rm g}$ [−]	isotropic growth parameters	NA	3., 12.8, 6.	adapted [2]
$\alpha_{\text{g-}\xi}$, $\beta_{\text{g-}\xi}$, $\gamma_{\text{g-}\xi}$ [−]	non-isotropic growth parameters	NA	0., 0., 0.	this work
$\alpha_{\text{g-}\epsilon}$, $\beta_{\text{g-}\epsilon}$, $\gamma_{\text{g-}\epsilon}$ [−]	non-isotropic growth parameters	NA	0., 0., 0.	this work

References

1. Voutouri C, Mpekris F, Papageorgis P, Odysseos AD, Stylianopoulos T. Role of Constitutive Behavior and Tumor-Host Mechanical Interactions in the State of Stress and Growth of Solid Tumors. PLoS ONE. 2014;9(8):e104717.

2. Wijeratne PA, Vavourakis V, Hipwell JH, Voutouri C, Papageorgis P, Stylianopoulos T, et al. Multiscale modelling of solid tumour growth: the effect of collagen micromechanics. Biomechanics and Modeling in Mechanobiology. 2015;p. 1–12.
