# Supplementary material for: A Validated Multiscale In-Silico Model for Mechano-sensitive Tumour Angiogenesis and Growth
Source: PLoS Comput Biol. 2017 Jan 26;13(1):e1005259. doi: 10.1371/journal.pcbi.1005259 (PMC5268362; doi:10.1371/journal.pcbi.1005259)
Supplement: S2 Table — List of model parameters associated with the Fluid Solver Module. Cells marked with an asterisk denote shared values for both tissue types. (PDF) [file pcbi.1005259.s024.pdf]

# SUPPORTING INFORMATION

## A Validated Multiscale In-silico Model for Mechano-sensitive Tumour Angiogenesis and Growth

Vasileios Vavourakis, Peter A. Wijeratne, Rebecca Shipley, Marilena Loizidou, Triantafyllos Stylianopoulos, David J. Hawkes

### Fluid mechanics model parameters

List of model parameters associated with the *Fluid Solver Module* (see Fig 3). Cells marked with an asterisk denote shared values for both tissue types.

| **Parameter** | **Description** | **Host** | **Tumour** | **Source** |
|---|---|---|---|---|
| [mm-Hg] | Inlet: prescribed pressure | 25. | * | [1] |
| [mm-Hg] | Outlet: prescribed pressure | 10. | * | [1] |
| $\mu_{\mathrm{B}}$ [mm-Hg s] | blood viscosity | $3.e{-}5$ | * | [2] |
| $S_{\mathrm{vsc}}$ [cm$^{-1}$] | vascular density | 70. | 200. | [3–5] |
| $K_{\mathrm{int}\text{-}\{\mathrm{T}\vert\mathrm{H}\}}$ [cm$^2$(mm-Hg s)$^{-1}$] | hydraulic conductivity of the interstitium | $8.51e{-}9$ | $2.5e{-}7$ | [6–8] |
| $\gamma_{\mathrm{p}}$ [–] | blood vessel wall pores fraction | $1.296e{-}5$ | $1.008e{-}2$ | from [9] and adapted [10, 11] |
| $\sigma_{\mathrm{o}}$ [–] | average osmotic reflection coefficient | 0.91 | $8.7e{-}5$ | [11–13] |
| $\pi_{\mathrm{vsc}}$ [mm-Hg] | plasma osmotic pressure | 20. | 19.3 | [11, 14] |
| $\pi_{\mathrm{int}}$ [mm-Hg] | interstitial fluid osmotic pressure | 10. | 17.3 | [10, 11] |

# References

1. Wu J, Xu S, Long Q, Collins MW, König CS, Zhao G, et al. Coupled modeling of blood perfusion in intravascular, interstitial spaces in tumor microvasculature. Journal of Biomechanics. 2008;41(5):996–1004.

2. Fung YC. Biomechanics: Motion, Flow, Stress and Growth. New York, NY: Springer; 1990.

3. Hilmas DE, Gillette EL. Morphometric analyses of the microvasculature of tumors during growth and after X-irradiation. Cancer. 1974;33(1):103–110.

4. Jain RK. Transport of molecules across tumor vasculature. Cancer Metastasis Rev. 1987b;6:559–594.

5. Soltani M, Chen P. Numerical Modeling of Fluid Flow in Solid Tumors. PLoS ONE. 2011;6(6):e20344.

6. Swabb EA, Wei J, Gullino PM. Diffusion and Convection in Normal and Neoplastic Tissues. Cancer Research. 1974;34(10):2814–2822.

7. Jain RK. Transport of molecules in the tumor interstitium: A review. Cancer Research. 1987a;47:3039–3051.

8. Netti PA, Berk DA, Swartz MA, Grodzinsky AJ, Jain RK. Role of Extracellular Matrix Assembly in Interstitial Transport in Solid Tumors. Cancer Research. 2000;60(9):2497–2503.

9. Rippe B, Kamiya A, Folkow B. Simultaneous measurements of capillary diffusion and filtration exchange during shifts in filtration-absorption and at graded alterations in the capillary permeability surface area product (PS). Acta Physiologica Scandinavica. 1978;104(3):318–336.

10. Baxter LT, Jain RK. Transport of fluid and macromolecules in tumors. I. Role of interstitial pressure and convection. Microvascular Research. 1989;37(1):77–104.

11. Jain RK, Tong RT, Munn LL. Effect of vascular normalization by antiangiogenic therapy on interstitial hypertension, peritumor edema, and lymphatic metastasis: Insights from a mathematical model. Cancer Research. 2007;67(6):2729–2735.

12. Ballard K, Perl W. Osmotic reflection coefficients of canine subcutaneous adipose tissue endothelium. Microvascular Research. 1978;16(2):224–236.

13. Curry FE. Handbook of Physiology, Section 2: The Cardiovascular System. Bethesda, MD: American Physiological Society; 1984. p. 309–374.

14. Brace RA, Guyton AC. Interaction of transcapillary Starling forces in the isolated dog forelimb. American Journal of Physiology - Heart and Circulatory Physiology. 1977;233(1):H136–H140.
