# Supplementary material for: A Validated Multiscale In-Silico Model for Mechano-sensitive Tumour Angiogenesis and Growth
Source: PLoS Comput Biol. 2017 Jan 26;13(1):e1005259. doi: 10.1371/journal.pcbi.1005259 (PMC5268362; doi:10.1371/journal.pcbi.1005259)
Supplement: S3 Table — List of model parameters associated with the Biochemical Solver Module. Cells marked with an asterisk denote shared values for both tissue types, while “NA” denotes non-applicable. (PDF) [file pcbi.1005259.s025.pdf]

# SUPPORTING INFORMATION

## A Validated Multiscale In-silico Model for Mechano-sensitive Tumour Angiogenesis and Growth

Vasileios Vavourakis, Peter A. Wijeratne, Rebecca Shipley, Marilena Loizidou, Triantafyllos Stylianopoulos, David J. Hawkes

### Biochemical model parameters

List of model parameters associated with the *Biochemical Solver Module* (see Fig 3). Cells marked with an asterisk denote shared values for both tissue types, while "NA" denotes non-applicable.

| Parameter | Description | Host | Tumour | Source |
|---|---|---|---|---|
| $\lambda_\tau$ [d$^{-1}$] | TAF production rate | NA | 9.996 | adapted [1] |
| $\delta_\tau$ [d$^{-1}$] | TAF decay rate | 0.0864 | $*$ | adapted [1] |
| $D_\tau$ [m$^2$ d$^{-1}$] | TAF diffusion coefficient | $0.999e-6$ | $*$ | [2] |
| [g cm$^{-3}$] | maximum reference TAF concentration in tumour | NA | $1.e-8$ | estimated [3] |
| $\lambda_\xi$ [d$^{-1}$] | $O_2$ supply per unit of capillary density/time | 0.288 | $*$ | adapted [1,4] |
| $\delta_\xi$ [d$^{-1}$] | tissue $O_2$ uptake | $0.138e-2$ | $1.725e-2$ | from [5] and adapted [1] |
| $D_\xi$ [m$^2$ d$^{-1}$] | $O_2$ diffusion rate | $3.455e-9$ | $*$ | [6] |
| $\xi$ [–] | $O_2$-related parameter regulating TAF production | NA | 0.6 | this work |
| $\lambda_{\mu\text{-c}}$ [d$^{-1}$] | tumour cells MDE production rate | NA | $4.32e-2$ | adapted [1] |
| $\lambda_{\mu\text{-v}}$ [d$^{-1}$] | tip-ECs MDE production rate | $0.864e-2$ | $*$ | adapted [7] |
| $\delta_\mu$ [d$^{-1}$] | MDE decay rate | 103.68 | $*$ | adapted [7,8] |
| $D_\mu$ [m$^2$ d$^{-1}$] | MDE diffusion coefficient | $7.344e-7$ | $*$ | adapted [7,9] |
| [Mol cm$^{-3}$] | maximum reference MDE molecular concentration | $1.e-13$ | $*$ | [10] |
| $\delta_\epsilon$ [d$^{-1}$] | ECM degradation rate | 6.912 | NA | adapted [7] |
| [g cm$^{-3}$] | reference ECM density (*wrt* collagen concentration) | 0.1 | NA | [3] |

# References


1. Wu M, Frieboes HB, McDougall SR, Chaplain MAJ, Cristini V, Lowengrub J. The effect of interstitial pressure on tumor growth: coupling with the blood and lymphatic vascular systems. Journal of Theoretical Biology. 2013;320:131–151.

2. Bray D. Cell Movements: From Molecules to Motility. 2nd ed. Garland Science; 2000.

3. Olsen L, Sherratt JA, Maini PK. A Mechanochemical Model for Adult Dermal Wound Contraction and the Permanence of the Contracted Tissue Displacement Profile. Journal of Theoretical Biology. 1995;177(2):113–128.

4. Valero C, Javierre E, García-Aznar JM, Gómez-Benito MJ. Numerical modelling of the angiogenesis process in wound contraction. Biomechanics and Modeling in Mechanobiology. 2013;12(2):349–360.

5. Vaupel P, Fortmeyer HP, Runkel S, Kallinowski F. Blood Flow, Oxygen Consumption, and Tissue Oxygenation of Human Breast Cancer Xenografts in Nude Rats. Cancer Research. 1987;47(13):3496–3503.

6. Wang CH, Li J. Three-dimensional simulation of IgG delivery to tumors. Chemical Engineering Science. 1998;53(20):1081–1089.

7. Wood LB, Ge R, Kamm RD, Asada HH. Nascent vessel elongation rate is inversely related to diameter in in vitro angiogenesis. Integrative Biology. 2012;4:3579–3600.

8. Anderson ARA. A hybrid mathematical model of solid tumour invasion: the importance of cell adhesion. Mathematical Medicine and Biology. 2005;22(2):163–186.

9. Karagiannis ED, Popel AS. Distinct modes of collagen type I proteolysis by matrix metalloproteinase (MMP) 2 and membrane type I MMP during the migration of a tip endothelial cell: Insights from a computational model. Journal of Theoretical Biology. 2006;238(1):124–145.

10. Milde F, Bergdorf M, Koumoutsakos P. A Hybrid Model for Three-Dimensional Simulations of Sprouting Angiogenesis. Biophysical Journal. 2008;95:3146–3160.
