# Supplementary material for: A Validated Multiscale In-Silico Model for Mechano-sensitive Tumour Angiogenesis and Growth
Source: PLoS Comput Biol. 2017 Jan 26;13(1):e1005259. doi: 10.1371/journal.pcbi.1005259 (PMC5268362; doi:10.1371/journal.pcbi.1005259)
Supplement: S4 Table — List of model parameters associated with the Vascular Network Module. Parameters with a star (⋆) correspond to non-perfused or hypo-perfused vessels, while those with a dagger (†) correspond to well-perfused vessels. The parameters with a double dagger (‡) denote the pre-set parameter values of the original vascular network. (PDF) [file pcbi.1005259.s026.pdf]

SUPPORTING INFORMATION

A Validated Multiscale In-silico Model for Mechano-sensitive Tumour Angiogenesis and Growth

Vasileios Vavourakis, Peter A. Wijeratne, Rebecca Shipley, Marilena Loizidou, Triantafyllos Stylianopoulos, David J. Hawkes

Vascular network model parameters

List of model parameters associated with the *Vascular Network Module* (see Fig 3). Parameters with a star ($\star$) correspond to non-perfused or hypo-perfused vessels, while those with a dagger (†) correspond to well-perfused vessels. The parameters with a double dagger (‡) denote the pre-set parameter values of the original vascular network.

Parameter	Description	Host	Tumour	Source
k_τ [m]	chemotaxis in angiogenesis	1.		this work
k_ϵ [m]	haptotaxis in angiogenesis	0.3		this work
k_m [Pa^{-1}]	mechanotaxis in angiogenesis	0.01		this work
$\tilde{R}$ [μm]	see Eq (17)	4.		[1]
$v_{\text{v-0}}$; $v_{\text{v-1}}$ [μm d^{-1}]		5.5; 479.7		[1]
$v_{\text{v-max}}$ [μm d^{-1}]		250.		adapted [1]
τ^* [–]	TAF threshold above which angiogenesis occurs	0.0125		this work
μ_D, σ_D [μm]	median and standard deviation of distance-related probability function $\mathcal{P}_\mathrm{D}$	$150. \pm 15^\star$/ $300. \pm 30^\dagger$		this work
μ_A, σ_A [d]	median and standard deviation of age-related probability function $\mathcal{P}_\mathrm{A}$	$0.5 \pm 0.25^\star$/ $1.25 \pm 0.25^\dagger$		this work
[μm]	minimum distance (between two different branches) to enforce anastomosis	40.		this work
R [μm]	capillary lumen radius of original vessels; range of R for tumour vessels	$80. \pm 0.8^\ddagger$; 5.—50.		[2,3]
h [μm]	capillary wall thickness of original vessels; range of h for tumour vessels	$5.5 \pm 0.055^\ddagger$; 1.49—4.01		[2,3]
r_p [nm]	capillary wall pore size of original vessels; range of r_p for tumour vessels	$5 \pm 0.02^\ddagger$; 300.—500.		[4,5]
e_c [–]	maximum (radial) strain of collapse for the capillary wall	92%		this work
$E_{\text{w-max}}$ [–]	range of maximum capillary wall modulus of rigidity	1.3—5.22		this work

References

1. Wood LB, Ge R, Kamm RD, Asada HH. Nascent vessel elongation rate is inversely related to diameter in in vitro angiogenesis. Integrative Biology. 2012;4:3579–3600.

2. Hashizume H, Baluk P, Morikawa S, McLean JW, Thurston G, Roberge S, et al. Openings between Defective Endothelial Cells Explain Tumor Vessel Leakiness. The American Journal of Pathology. 2000;156(4):1363–1380.

3. Morikawa S, Baluk P, Kaidoh T, Haskell A, Jain RK, McDonald DM. Abnormalities in Pericytes on Blood Vessels and Endothelial Sprouts in Tumors. The American Journal of Pathology. 2002;160(3):985–1000.

4. Sarin H. Physiologic upper limits of pore size of different blood capillary types and another perspective on the dual pore theory of microvascular permeability. Journal of Angiogenesis Research. 2010;2(1):1–19.

5. Chauhan VP, Stylianopoulos T, Martin JD, Popovic Z, Chen O, Kamoun WS, et al. Normalization of tumour blood vessels improves the delivery of nanomedicines in a size-dependent manner. Nature Nanotechnology. 2012;7(6):383–388.
